# VoiceS: voice quality after transoral CO_2_ laser surgery versus single vocal cord irradiation for unilateral stage 0 and I glottic larynx cancer—a randomized phase III trial

**DOI:** 10.1186/s13063-022-06841-5

**Published:** 2022-10-27

**Authors:** Philipp Reinhardt, Roland Giger, Eberhard Seifert, Mohamed Shelan, Elena Riggenbach, Dario Terribilini, Andreas Joosten, Daniel H. Schanne, Daniel M. Aebersold, Peter Manser, Matthias S. Dettmer, Christian Simon, Esat M. Ozsahin, Raphaël Moeckli, Andreas Limacher, Francesca Caparrotti, Deepa Nair, Jean Bourhis, Martina A. Broglie, Abrahim Al-Mamgani, Olgun Elicin

**Affiliations:** 1 Department of Radiation Oncology, Bern University Hospital and University of Bern, Inselspital, Freiburgstrasse 18, 3010 Bern, Switzerland; 2 Department of Oto-Rhino-Laryngology, Head and Neck Surgery, Bern University Hospital and University of Bern, Inselspital, Freiburgstrasse 18, 3010 Bern, Switzerland; 3 Division of Phoniatrics, Department of Oto-Rhino-Laryngology, Head and Neck Surgery, Bern University Hospital and University of Bern, Inselspital, Freiburgstrasse 18, 3010 Bern, Switzerland; 4 Division of Medical Radiation Physics and Department of Radiation Oncology, Inselspital, Bern University Hospital and University of Bern, Freiburgstrasse 18, 3010 Bern, Switzerland; 5 Department of Pathology, Klinikum Stuttgart, Kriegsbergstraße 60, 70174 Stuttgart, Germany; 6 Department of Pathology, University of Bern, Murtenstrasse 31, 3010 Bern, Switzerland; 7 Department of Otolaryngology - Head and Neck Surgery, CHUV University of Lausanne, Rue du Bugnon, 2, 1011 Lausanne, Switzerland; 8 Department of Radiation Oncology, Lausanne University Hospital and Lausanne University, Rue du Bugnon, 21, 1011 Lausanne, Switzerland; 9 Institut of Radiation Physics, Lausanne University Hospital and Lausanne University, Rue du Grand-Pré 1, 1007 Lausanne, Switzerland; 10 Clinical Trials Unit Bern, University of Bern, Mittelstrasse 43, 3012 Bern, Switzerland; 11 Department of Radiation Oncology, Genève University Hospital, Rue Gabrielle-Perret-Gentil 4, 1205 Genève, Switzerland; 12 Department of Head Neck Surgical Oncology, ACTREC, Tata Memorial Centre, Homi Bhabha National Institute, Mumbai, 400012 India; 13 Department of Head and Neck Surgery, University Hospital of Zurich, Rämistrasse 100, 8091 Zürich, Switzerland; 14 Department of Radiation Oncology, Netherlands Cancer Institute/Antoni van Leeuwenhoek, 1066 Amsterdam, CX Netherlands

**Keywords:** Glottic cancer, Larynx, Radiotherapy, Vocal cord irradiation, Transoral CO_2_-laser surgery, Randomized controlled trial

## Abstract

**Background:**

Surgery and radiotherapy are well-established standards of care for unilateral stage 0 and I early-stage glottic cancer (ESGC). Based on comparative studies and meta-analyses, functional and oncological outcomes after both treatment modalities are similar. Historically, radiotherapy (RT) has been performed by irradiation of the whole larynx. However, only the involved vocal cord is being treated with recently introduced hypofractionated concepts that result in 8 to 10-fold smaller target volumes. Retrospective data argues for an improvement in voice quality with non-inferior local control. Based on these findings, single vocal cord irradiation (SVCI) has been implemented as a routine approach in some institutions for ESGC in recent years. However, prospective data directly comparing SVCI with surgery is lacking. The aim of VoiceS is to fill this gap.

**Methods:**

In this prospective randomized multi-center open-label phase III study with a superiority design, 34 patients with histopathologically confirmed, untreated, unilateral stage 0-I ESGC (unilateral cTis or cT1a) will be randomized to SVCI or transoral CO_2_-laser microsurgical cordectomy (TLM). Average difference in voice quality, measured by using the voice handicap index (VHI) will be modeled over four time points (6, 12, 18, and 24 months). Primary endpoint of this study will be the patient-reported subjective voice quality between 6 to 24 months after randomization. Secondary endpoints will include perceptual impression of the voice via roughness – breathiness – hoarseness (RBH) assessment at the above-mentioned time points. Additionally, quantitative characteristics of voice, loco-regional tumor control at 2 and 5 years, and treatment toxicity at 2 and 5 years based on CTCAE v.5.0 will be reported.

**Discussion:**

To our knowledge, VoiceS is the first randomized phase III trial comparing SVCI with TLM. Results of this study may lead to improved decision-making in the treatment of ESGC.

**Trial registration:**

ClinicalTrials.gov NCT04057209. Registered on 15 August 2019. Cantonal Ethics Committee KEK-BE 2019-01506

**Supplementary Information:**

The online version contains supplementary material available at 10.1186/s13063-022-06841-5.

## Background

Head and neck squamous cell carcinoma (HNSCC) is the 6th most common type of cancer worldwide [1]. Of all head and neck cancers, approximately 30% originate from the larynx, resulting in 52000 newly diagnosed patients annually in Europe [2]. About 50–60% of laryngeal squamous cell carcinomas arise from the glottic region [3] and over 80% of these patients present in an early stage [4]. The larynx has an important role for voice, coordination of deglutition, and respiration. Therefore, the treatment aim of laryngeal cancer is not only the achievement of maximal disease control, but also the maintenance of function. Transoral CO_2_-Laser Microsurgical Cordectomy (TLM) and radiotherapy (RT) are well-established standard treatment modalities for unilateral ESGC (stage 0 and I) [5, 6]. Based on several clinical trials and meta-analyses, functional and oncological outcomes (local control (LC) and overall survival (OS)) after both treatment modalities are similar [7–11].

Treatment options vary remarkably in different countries and among institutions. Especially in the case of unilateral ESGC, other factors such as voice quality, cultural and socio-economic factors, and patients’ preferences are routinely considered. RT and TLM lead to similar outcomes, but differ significantly in treatment schedule. Surgery is performed in one day, usually followed by a very short hospital stay (1–2 nights). In contrast, RT is applied in daily fractions over the course of 4 to 7 weeks to the whole larynx [12], depending on institutional dosing and fractionation schemas. Post-treatment follow-up schedules are identical for both strategies. Among the most relevant treatment sequelae are voice quality and hoarseness due to altered cord volume, motion, and anatomy with impaired vocal fold closure. Several studies have shown better voice quality after RT compared to TLM [13–16], whereas other reports have revealed no significant differences [17–19], potentially because of heterogeneity in voice analysis techniques.

Recently, a new technique with advanced image guidance and modulated fields was introduced which allows the limitation of the treated volume to the involved vocal cord and results in an 8- to 10-fold reduction of target volumes compared to whole- larynx irradiation. Treatment is reduced to 16 single daily fractions with a higher dose per fraction (hypofractionated RT) [20–25]. Al-Mamgani et al. compared in a retrospective study SVCI with the results of a historical cohort treated with whole-larynx RT in the same institution [21]. Voice handicap index (VHI) was significantly superior to conventional RT at all time points beginning from the 6th week after SVCI. Moreover, a comparable local control with SVCI (100%) vs. 3D-conformal RT (92%) was reported at two years (*p*=0.24). Based on these results, SVCI has been implemented as a standard approach for ESGC in various radiation oncology centers in recent years.

In conclusion, long-term voice quality and oncologic outcome are comparable with both TLM and whole-larynx RT. Furthermore, SVCI possibly offers a superior long-term voice quality with at least an equal oncologic outcome compared to traditional RT. Based on that the aim of VoiceS is to compare SVCI to TLM with the focus on patient-reported voice quality.

Here, the summary of the protocol is presented. The full protocol is provided as supplementary material (see Additional file 1).

## Methods/design

The VoiceS protocol was constructed by using the SPIRIT reporting guidelines (see Additional file 2) [26].

### Regulatory approval

The study is listed in ClinicalTrials.gov and in the Swiss National Clinical Trials Portal (NCT04057209) after approval by the local ethics committee (Cantonal Ethics Committee Bern/ Switzerland, KEK-BE 2019-01506). Sponsor-Investigator and trial statistician have approved the protocol version 1.4 (01.12.2021) and confirm hereby to conduct the study according to the protocol, current version of the World Medical Association Declaration of Helsinki, ICH-GCP guidelines, and the local legally applicable requirements.

### Study population

#### Inclusion criteria

1. ECOG performance status 0–1 at the time of registration
2. ≥18 years of age
3. Baseline assessments and documentation of voice quality by means of VHI, JS, RBH, GNE, SPR
4. Histopathological confirmed, previously untreated stage 0 or I ESGC (unilateral cTis or cT1a) based on the UICC staging system (8th edition)
5. History and physical examination by treating physician (head and neck surgeon and radiation oncologist) within 28 days prior registration
6. The patient must be expected to withstand both study interventions
7. The patient must have undergone panendoscopy with assessment for the feasibility of transoral exposure for resection. Patients without feasible exposure are not eligible
8. Localization of the tumor should allow resection with a minimum of 2 mm macroscopic margin without extension to the contralateral vocal fold, without partial resection of the arytenoid cartilage, and without resection of parts of thyroid cartilage (Cordectomy Type I-IV according to the classification of the European Laryngological Society [27])
9. Hemoglobin ≥10 g/dL or 6.2 mmol/L (Note: The use of transfusion to achieve Hgb ≥10 g/dL is acceptable) within the 28 days prior to accrual
10. Women with childbearing potential and using effective contraception, and not pregnant and agree not to become pregnant during participation in the trial and 30 days after RT. A negative pregnancy test before inclusion (within 28 days) into the trial is required for all women with childbearing potential. Men agree not to father a child during participation in the trial and 30 days after RT.
11. Written informed consent, signed by the patient and the investigator.

#### Exclusion criteria

1. Infection hampering voice quality at the time of voice assessment
2. Involvement of the anterior commissure by the tumor
3. Previous oncologic surgery with curative intent (exception: excisional biopsies resulting in unacceptable close R0 or R1/R2 margins may be included) or RT to the larynx
4. Synchronous or previous malignancies. Exceptions are treated basal cell carcinoma or SCC of the skin, or in situ carcinoma of the cervix uteri, low-risk prostate cancer or breast with a cancer-free follow-up time of at least 3 years, or other previous malignancy with a progression-free interval of at least 5 years
5. Co-existing disease prejudicing survival (expected survival less than 6 months)
6. Active bacterial or fungal infection requiring intravenous antibiotics at the time of registration
7. History of any voice disorders (not related to the ESGC) lasting longer than 3 weeks
8. Illness requiring hospitalization or precluding study therapy within 28 days before registration
9. Presence of any psychological, familial, sociological, or geographical condition potentially hampering compliance with the study protocol and follow-up schedule; those conditions should be discussed with the patient before registration in the trial

### Recruitment and screening

Patient registration/randomization will only be accepted from authorized investigators. Prior to registration, the following steps have to be taken:

- Fill in the patient screening (used for monitoring potentially eligible patients), enrollment and identification lists.
- Check the eligibility criteria
- Obtain signed and dated written informed consent from the patient prior to any protocol-specific procedure according to ICH/GCP and local guidelines.
- Patients must complete the phoniatric assessments per protocol

The trial is open and currently accruing since 20 November 2019. Approximate recruitment will be completed at 01 May 2025. No special strategies for recruitment are intended.

### Study design and statistical considerations

**Fig. 1:**
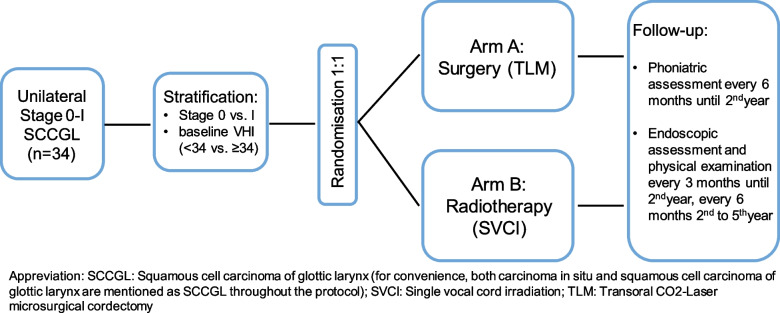
VoiceS study design

This is a prospective randomized multi-center open-label comparative phase III study with a superiority design. Patients fulfilling the eligibility criteria are randomized using a 1:1 ratio in the treatment arms TLM and SVCI (Fig. 1). Stratification factors are stage 0 vs. I and baseline VHI <34 vs. ≥34.

Based on the sample size calculation, 34 patients (17 per group) are needed to detect a difference in VHI at a two-sided alpha level of 0.05 with a power of 80%. Due to obvious differences between surgery and RT, it is not feasible to have a blinded design in this study setting.

### Outcomes

#### Primary endpoint

- Average of the VHI assessed at 6, 12, 18, and 24 months

#### Secondary endpoints

- VHI separately assessed at 6, 12, 18, and 24 months
- Perceptual impression of the voice via roughness – breathiness – hoarseness (RBH) assessment at 6, 12, 18, and 24 months
- Quantitative characteristics of voice by means of Jitter and Shimmer (JS), glottal-to-noise excitation ratio (GNE), and singing power ratio (SPR), which will be assessed at 6, 12, 18, and 24 months
- Loco-regional control at 2 and 5 years
- Treatment toxicity at 2 and 5 years based on CTCAE v.5.0

### Arm A: transoral CO_2_-laser microsurgical cordectomy (TLM)

TLM is performed using a CO_2_ laser coupled to an operative microscope. The type of cordectomies (I-IV) is defined according to the classification of the European Laryngological Society [26] and chosen to provide a complete removal of the primary lesion with negative margins. Surgery is performed within 3 weeks after randomization and not more than 6 weeks after panendoscopy. The extent of the cordectomy must include a complete anterior, posterior, inferior, and supero-lateral mucosal, and deep soft tissue margin. Positive margin is defined as tumor contact with resection margins.

Transoral re-resections within 4 weeks are required in case of R1 or close-margin (for Tis: 0 mm to <0.5 mm; for T1a: 0 mm to <1 mm) to convert the patient to an R0 status. TLM. Day 0 is the date of surgery. Surgical adverse events (AE) are recorded using the Clavien-Dindo classification of surgical complications [28].

**Fig. 2:**
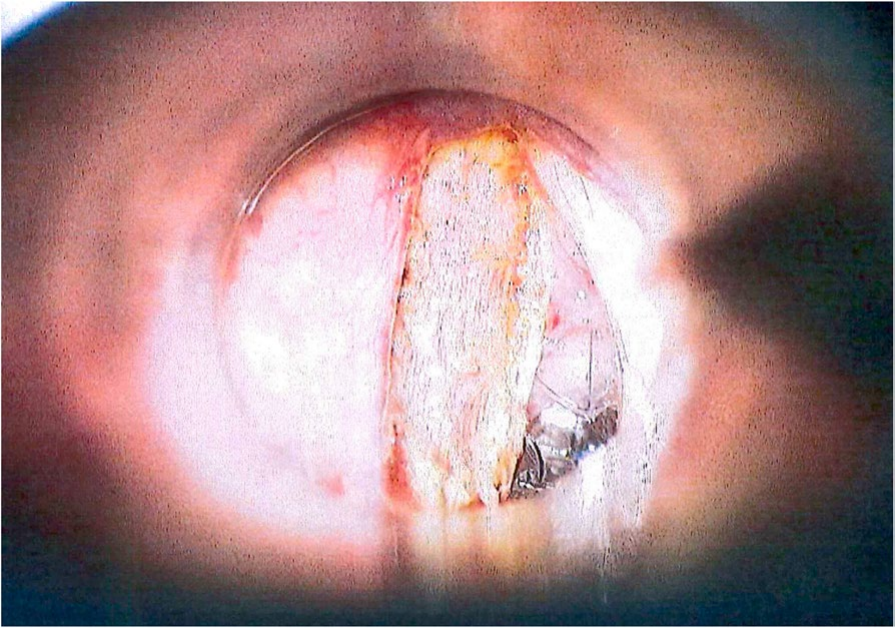
Endoscopic view during a type II cordectomy of the left side

**Fig. 3:**
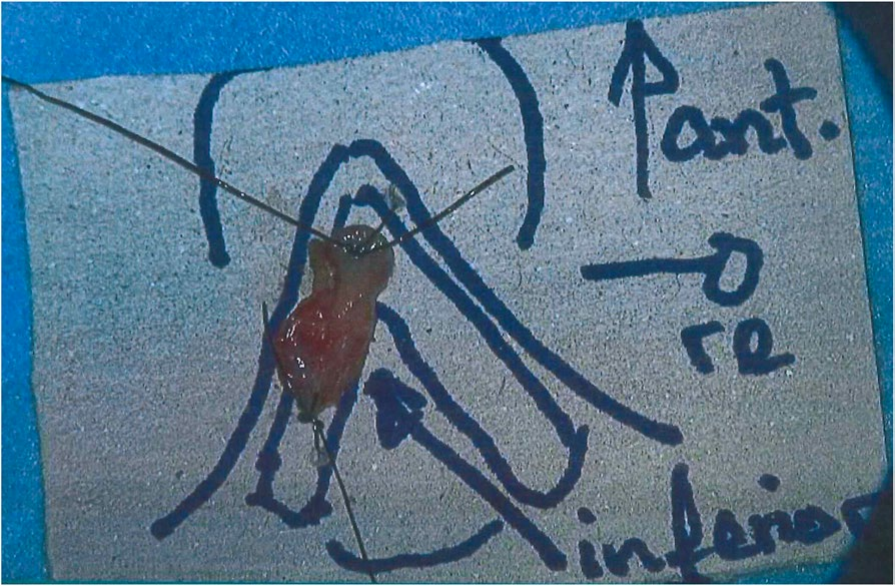
Formalin-fixed cordectomy specimen

Surgical technique in all participating centers is standardized. Margin status, in case of positive or close margins re-resection protocols, pathology reports of follow-up interventions, and surgical complications are subject to quality assurance (QA). The resection is adapted by the surgeon as per local practice but must allow the identification of all margins (Figs. 2 and 3).

The specimen must be formalin-fixed as quickly as possible within 2 h and subsequently paraffin-embedded within 48 h according to standard procedure. For each participating center, a prospective central surgical and pathology QA is performed. Acceptable minor variations (e.g., suboptimal resection with close margin), acceptable major deviations, and unacceptable deviations/protocol violations (e.g., positive margin without re-resection within 4 weeks or adjuvant radiotherapy within 5–6 weeks after the last surgical intervention, bleeding requiring operative control exceeding 20% of the approximated total blood volume) are determined. Quality indicators include margin status (clear vs. positive/close), minimal distance from tumor to surgical margin both mucosal and deep, type of peri- and postoperative complications, and frequency of revision procedures. Further details are provided in the full protocol (see Additional file 1).

### Arm B: single vocal cord irradiation (SVCI)

A volumetric treatment planning computed tomography (CT) is required to define the target volumes. The treatment planning CT scan must be performed with an immobilization device (thermoplastic masks covering at least the head and neck area) in place and in the treatment position. A respiratory-gated 4D-CT scan with 1 mm slice thickness is acquired. The patients are simulated and treated in supine position with arms on the side of the trunk, positioned and fixated according to the routines at each treatment center.

Treatment planning is performed using a state-of-the-art treatment planning system (TPS) which permits an optimization of dose-volume parameters for the planning target volume (PTV) and organs at risk (OAR). Gross tumor volume (GTV) and clinical target volume (CTV) are delineated. CTV is equal to GTV with an isotropic margin of 3 mm or the entire ipsilateral vocal cord (in case the GTV cannot be clearly delineated). The use of other imaging modalities (e.g., magnetic resonance imaging) to define the GTV is left at the decision of the responsible radiation oncologist in each center. In the axial plane, after the extension of the GTV, CTV must be adapted to anatomical structures. The PTV is defined by expansion of the CTV with the following margins left–right and anterior–posterior: 3 mm, cranial–caudal: 5 mm. All OAR are delineated according to the international consensus guidelines by Brouwer et al. [29]. The swallowing muscles (cricopharyngeous muscle, middle constrictor muscle, and inferior constrictor muscle) are delineated as defined by Christianen et al. [30].

**Fig. 4:**
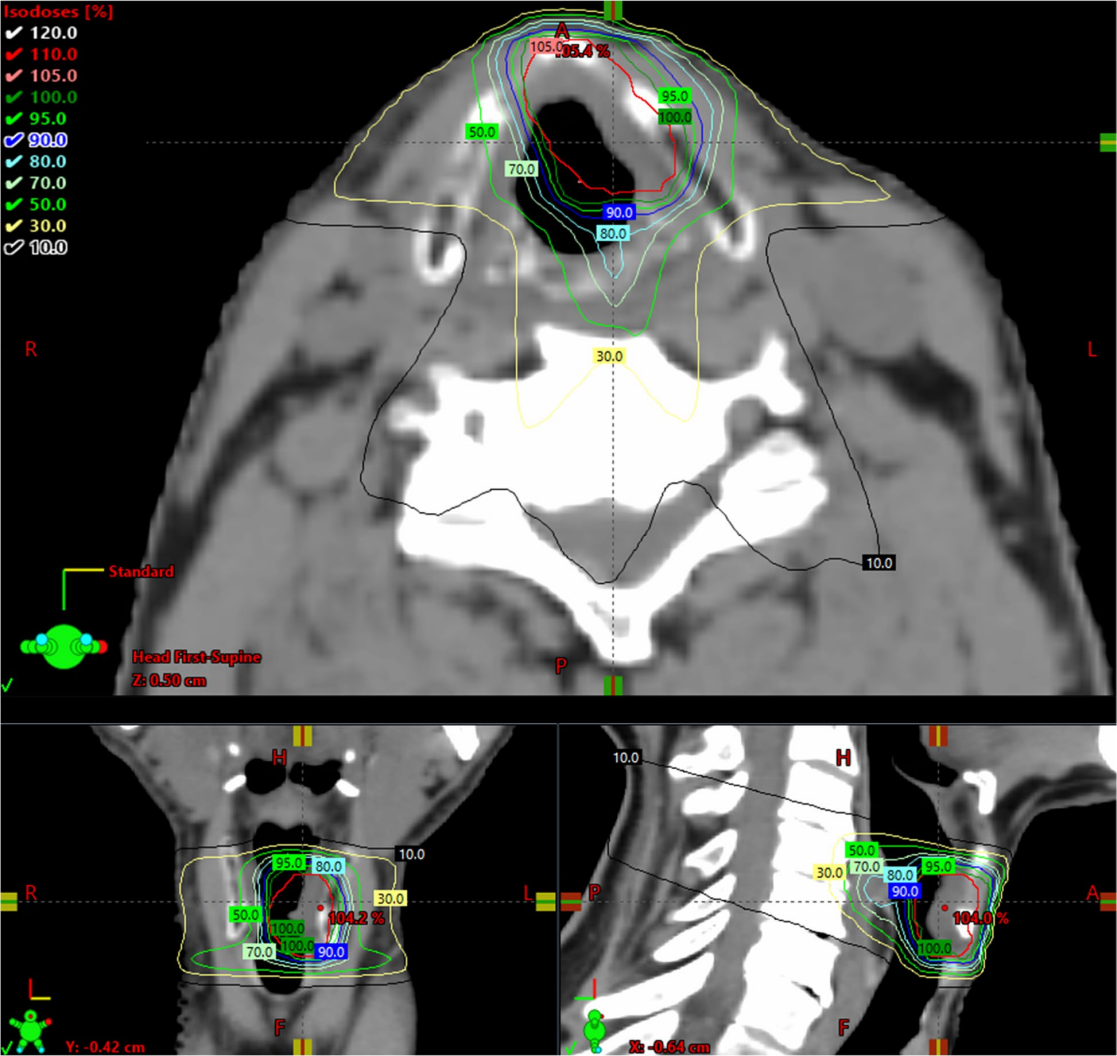
Example treatment plan with IMRT (five static fields)

**Fig. 5:**
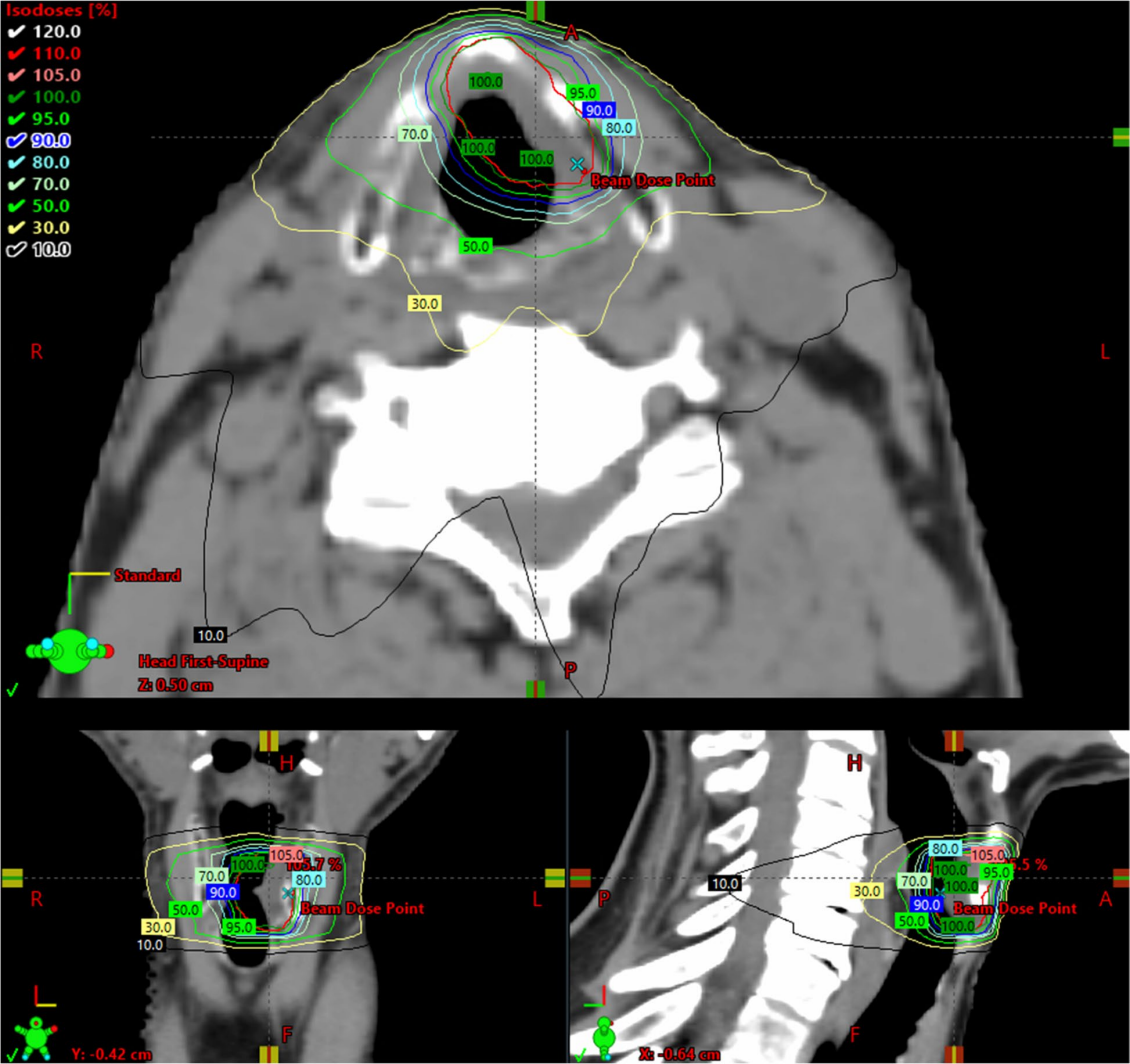
Example treatment plan with VMAT

Objectives for the PTV are its encompassment by at least 95% of the prescribed dose and a maximum dose, D_0.03cc_ , < 107%. Dose is delivered in 16 sequential single-day fractions (3.63 Gy each) on 5 days per week (16 x 3.63 = 58.08 Gy; EQD2 _α/β=10 Gy_ = 66 Gy, EQD2 _α/β=3 Gy_ = 77 Gy). A five to nine static-field IMRT plan (Fig. 4) and a volumetric modulated arc therapy (VMAT) plan (Fig. 5) are generated. The best plan in terms of dose constraints and objectives, at the discretion of the treating physician, is then applied.

The image-guided radiotherapy (IGRT) protocol mandates pre- and post-treatment controls of the positioning. Alternatively, the use of MRI-mounted irradiation equipment is also allowed.

The working group for radiotherapy quality assurance (RT-QA group) consists of all panel members for radiotherapy and radiotherapy quality assurance (QA). Before the start of the trial, each participating institution performs a two-step dummy run. In the first step, each center has to delineate target volumes and OAR according to the study protocol on CT images of a HNSCC patient made available by the RT-QA group. The physicians and the medical physicists from the RT-QA group review the contours. Major deviations require revision. The second step involves the generation of the RT plan per protocol. Additionally, a patient-specific pretreatment QA verification for the generated RT plan is performed and documented. Major deviations are communicated and a revised version of the treatment plan has to be submitted until no major deviations persist. After the treatment of the first two patients in Arm B (radiotherapy) in each center, an on-site or online monitoring by the RT-QA team is carried out to perform an offline review of the IGRT of these two patients’ treatment courses. Further details are provided in the full protocol (see Additional file 1).

### Assessment of outcomes

The time points of each assessment are provided in the following summary flowchart (see Table 1). No study-specific concomitant interventions are planned. However, symptom-oriented medications are allowed (e.g., pain medications to alleviate post-operative pain, steroids to alleviate laryngeal edema during radiotherapy) by the decision of the treating physician.

**Table 1:** Summary flowchart

Study periods	Screening and enrollment	Treatment period	Follow-up until 60 months after enrollment
Time unit	Days	Weeks	Months
Patient information and informed consent	− 28–0		
Medical history and demographics	− 28–0		
Panendoscopy, assessment of exposure, and staging according to institutional local standards	− 28–0		
Eligibility criteria check	− 28–0		
ESGC-oriented physical examination	− 28–0		
VHI^a^, RBH, GNE, SPR, JS	− 28–0		(1.5)^a^, 6, 12, 18, 24 (+/− 15 days)
Enrollment and randomization^b^	0		
Endoscopic assessment of the index tumor, symptom-oriented physical examination	− 28–0		Every 3 months (+/− 15 days) until 24th month every 6 months (+/− 15 days) between 24th and 60th months
Evaluation of actual smoking status and toxicity according to CTCAE v.5.0 (see Additional file)	− 28–0 (baseline smoking status and toxicity^c^)	weekly during radiotherapy and within one week of surgery: no need to document except for SAEs	
Pregnancy test for women with child-bearing potential	− 28–0	3–7^d^	Until 3rd month^d^
Arm A: transoral CO_2_-laser microsurgical cordectomy^e^		day of surgery +/− day of re-resection	
Arm B: single vocal cord irradiation		3 (16 fractions)	

*GNE* glottal-to-noise excitation ratio, *JS* Jitter and Shimmer, *RBH* roughness – breathiness – hoarseness, *SAEs* serious adverse events, *ESGC* early-stage glottic cancer, *SPR* singing power ratio, *VHI* voice handicap index

^a^VHI at 6 weeks after the end of treatment (regardless of study arm) will be assessed to evaluate the need for phoniatric rehabilitation, but not as a study-specific outcome measure (i.e., primary endpoint) to avoid lead time bias. Patients with a VHI > 14 will be advised to undergo a phoniatric rehabilitation, but the examiner should not try to persuade the patient. Similarly, any VHI >14 at any follow-up time point or patients’ requests justify the need for phoniatric rehabilitation, which will be recommended to the patients regardless of study arm. No need to record the VHI in the CRFs

^b^Even if done on the same day, patient enrollment shall be done after the completion of the necessary documentation

^c^The baseline toxicity scores and smoking status may be documented after the accrual but it must be done before the start of trial treatment

^d^If clinically indicated

^e^The treatment shall start within 28 days post-accrual

### Assessment of primary outcome

The subjective voice quality is assessed by using the VHI [31]. The 30-item questionnaire is provided in different languages and affected patients describe the degree to which their voice restricts their everyday life. The higher the score (0–120), the greater is their subjectively experienced handicap (score 0–14 = no; 15–28 = mild; 29–50 = moderate; 51–120 = severe handicap). For recording the VHI at the foreseen visits, the open-source software DigitalVHI by Christian Herbst et al. [32] is used. In case of technical difficulties, paper forms with identical content and layout are also allowed.

### Assessment of secondary outcomes

Secondary endpoints are assessed by using the parameters roughness, breathiness, and hoarseness (RBH). By means of reading the phonetically balanced text “The Rainbow Passage” in English or “Die Sonne und der Wind” in German, French, Spanish, Portuguese and Italian, the speaking voice is blindly assessed by the phoniatricians according to the parameters RBH, using the scale of 0: normal, 1: mild, 2: moderate, 3: severe. In this study, the assessment is carried out blind. The quantitative characteristics of voice are blindly investigated by the following parameters: Jitter and shimmer (JS) [33], glottal-to-noise excitation ratio (GNE), and singing power ratio (SPR). Blinded analysis of GNE and SPR will be done by recording of vocal samples and subsequent evaluation using the open-source freeware software Praat (http://www.fon.hum.uva.nl/praat/ by Paul Boersma and David Weenink - Phonetic Sciences, University of Amsterdam Spuistraat 210 1012VT Amsterdam, Netherlands).

### Toxicity and oncological endpoints

Oncological endpoint is the loco-regional control of ESGC which is defined as the time between randomization and biopsy-proven HNSCC recurrence. The severity of all AEs (serious and non-serious) in this trial should be graded using CTCAE v.5.0. A serious adverse event (SAE) is defined as any untoward medical occurrence or effect in a patient, whether or not considered related to the protocol treatment, that:

- Results in death;
- Is life-threatening (i.e., an event in which the subject was at risk of death at the time of event; it does not refer to an event which hypothetically might have caused death if it was more severe);
- Requires inpatient hospitalization or prolongation of existing patient hospitalization;
- Results in persistent or significant disability or incapacity;
- Is a congenital anomaly or birth defect; and
- Is a medically important event or reaction.

Safety endpoints will be reported only at two time points (2 and 5 years). Toxicity is systematically assessed during the treatment, every 3 months until 24 months, and every 6 months between the 24th and 60th months. AE of grades 1 to 2 (CTCAE v.5.0) between the recruitment and before the 6th month visit do not need to be reported in the study-specific documents and will be managed according to institutional local standards. Any AE of grade 3 and above is considered as a SAE. From the time of patient’s registration in the study, any SAE, or follow-up to a SAE, including death due to any cause, that occurs to any subject must be reported within 24 h to the Sponsor if it is the result of a protocol-specified intervention/procedure.

### Assessments in participants who prematurely stop the study

If patients are withdrawn because of an AE, they will undergo physical examination and additional tests (e.g., laboratory testing) according to the nature of the AE. Unless the AE causes a contraindication for the planned treatment, it shall be performed as planned according to the tumor board decision. A last examination for possible (additional) toxicities shall be carried out at the time of the (written) withdrawal. The follow-up period ends after this last examination or when patients are dismissed from the hospital after the treatment of the AE, whichever happens last.

### Statistical methods

#### Hypothesis and determination of sample size

Sample size calculation is based on the primary outcome (VHI at 6 to 24 months). We hypothesized that there is a difference between TLM and SVCI regarding VHI. A difference of 8 points between the two groups is regarded as clinically relevant and a conservative standard deviation of 8 points is assumed. Based on a two-sample means test, 34 patients (17 per group) are needed to detect a difference in VHI at a two-sided alpha-level of 0.05 with a power of 80%. The average change from baseline to the four time points (6, 12, 18, and 24 months) will be modeled and additionally adjusted for the baseline VHI to yield more power. No statistical criteria for the termination of the trial are defined. No interim analysis for futility or safety will be performed.

#### Randomization and stratification

Executing a probabilistic minimization technique for random treatment allocation with a 1:1 ratio between the two treatment arms, computer-based treatment randomization is performed in dependence of the two predefined stratification factors tumor stage (stage 0 vs. I) and VHI at baseline (<34 vs. ≥34). Allocation will be done via a dedicated website within the clinical trial management system that also contains the electronic case report forms.

#### Planned analyses

Primary analysis will follow the intention-to-treat (ITT) principle. VHI collected over 24 months will be analyzed using a repeated-measures, linear mixed-effects model with a random intercept for the patients and fixed effects for the baseline value, the treatment group, the time points (categorical), interaction terms between the treatment group and time points, and randomization stratification factors. We will present the mean VHI difference between the two groups with associated 95% confidence intervals and *p*-values over all time points (primary outcome) as well as separately for each time point (secondary outcomes) from this model. JS, GNE, and SPR will be measured on a continuous scale and, therefore, will be analyzed using the same approach as for VHI. Jitter and shimmer are expected to show a log-normal distribution [33] and will therefore be log-transformed before the analysis. If the distributions of the other two outcomes should severely deviate from a normal distribution, these measures will also be transformed in an adequate way, e.g., by log-transformation. If normality cannot be achieved, measures will be analyzed using a non-parametric Wilcoxon-rank sum test, separately for each time point. Roughness, breathiness, and hoarseness are each assessed on an ordinal scale ranging from 0 to 3. These measures will be dichotomized into 2/3 versus 0/1 and analyzed using mixed-effects logistic regression with a random intercept for the patients and fixed effects for the treatment group, the time points (categorical), interaction terms between the treatment group and time points, and randomization stratification factors. We will present the odds ratio with associated 95% confidence intervals and *p*-values over all time points as well as separately for each time point from this model. The time-to-event outcome loco-regional control will be evaluated using Kaplan-Meier curves and a Cox- regression model adjusted for the randomization stratification factors. Treatment toxicity at 2 and 5 years will be summarized descriptively for each group, showing the overall number of events as well as the number and percentage of patients with events. Patients will be censored for toxicity assessment at the time of loco-regional and/or distant tumor recurrence.

In a secondary analysis, only patients are evaluated using the per-protocol (PP) patient set. Moreover, we will perform subgroup analyses according to tumor stage (stage 0 vs. I), and VHI at baseline (<34 vs. ≥34). For RBH, we will also consider a proportional-odds logistic regression of the original ordinal outcome. Since the assessment is qualitative and performed by different raters, we will also test for heterogeneity between the rating phoniatricians (blinded to the allocated treatment arm) and include a random intercept for raters in logistic models, if necessary. Toxicities in both arms will be described and compared with TAME [34] methodology.

#### Handling of missing data and drop-outs

We expect that all randomized patients have complete baseline data. All patients that have at least one outcome assessment can be considered in repeated-measures analyses. Models will implicitly correct for missing data based on the missing at random mechanism. If there are patients with no outcome data at all, we will perform multiple imputation. Details will be described in the statistical analysis plan (SAP). For the time-to-event analysis, patient dropouts will be accounted for by censoring.

## Discussion

TLM and RT are well-established standard treatment modalities for unilateral stage 0-I ESGC. In international cancer treatment guidelines, both treatment modalities are considered as similar for therapy of ESGC [5, 6]. Based on clinical trials and meta-analyses, functional and oncological outcome (overall survival and local control) between both treatment modalities was reported without statistical significance [7–11]. Kim et al. investigated the outcome of 14,498 patients with early-stage glottic cancer (ESGC) treated with surgery or RT (two different radiation dose fractionations: normofractionation and hypofractionation) [35]. Results demonstrated a 5-year OS for surgery and RT of 77.5% and 72.6% (*P* < 0.0001), but there was no significant difference in OS (72.6% vs 75.1%; *P* = 0.154) between patients undergoing surgery or hypofractionated RT (63-67.5 Gy in 28–30 fractions). Valculik et al. [36] published conflicting results in a systematic review and meta-analysis. Sixteen studies that compared the oncologic outcome in patients with T1 glottic carcinoma treated with TLM or RT were included. OS, disease-specific survival, and laryngeal preservation were better after the treatment with TLM. Because of the lack of randomized control trials, the results must be regarded with caution.

Neither surgery nor RT seems to be superior in terms of oncologic outcome. Voice quality is one of the key factors for evaluating functional outcomes after treatment of ESGC. A relatively small study by Mehel et al. [37] investigated voice quality in early-stage glottic carcinoma (Tis, T1a, T1b cN0) by comparing RT and TLM. No significant differences in the endpoints VHI scores and grade, roughness, breathiness, asthenia, and strain score (GRBAS) were found between both treatment modalities. The authors conclude that the choice of the treatment in the included Tis-T1a/b N0 ESGC should be considered by weighting factors like age, occupation, comorbidities, costs of treatment, hospital stay, and patients’ preference.

A meta-analysis published by Mendenhall et al. [38] compared TLM, open partial surgery, and RT in patients with ESGC (T1–T2 N0). Based on 10 included studies, there were no differences in LC, laryngeal preservation, and survival rates with these three modalities. Especially in limited T1a glottic carcinomas, the outcome of voice quality was similar after TLM and RT. However, the poorest voice quality was reported after an open partial laryngectomy. Another meta-analysis performed by Greulich et al. [39], focused on patient-reported voice quality after TLM in comparison to RT in stage I glottic carcinoma. Including eight retrospective publications without randomization, no significant difference in VHI after both treatment modalities was found. In this analysis, six studies resulted in a similar VHI after treatment, and two studies favored RT over surgery.

So far, no randomized trial has compared VHI in response to different treatments. The only prospective randomized study with the endpoint voice quality by Aaltonen et al. was closed prematurely due to low accrual [16]. Evaluation of included patients showed a comparable voice quality and oncological outcome between TLM and normofractionated RT with conventional whole-larynx volumes. Nevertheless, results suggested a less breathy voice after irradiation. Due to the unavailability of the VHI in the Finnish language at the time point of randomization, this endpoint could not be investigated. Recently, long-term results after a median follow-up of 6.6 years were published and reported a similar 5-year OS (TLM: 87%, RT: 92%) and larynx preservation rates TLM: 97%, RT: 92%) [40].

Inconsistent results of voice quality between both treatment modalities were reported extensively. However, several studies revealed no significant differences in voice analysis, either due to heterogeneity or the absence of a meaningful difference [17–19]. In contrast, some studies found a better voice quality after RT compared with TLM [13–16], whereas others reported superior voice quality outcomes after surgery [41, 42]. Additionally, there is debate about the ideal TLM technique. Strieth et al. showed in a randomized clinical trial that potassium-titanyl-phosphate (KTP) laser could be superior in terms of voice quality and LC comparing with standard CO_2_ laser [43]. Nevertheless, most of the literature consists of retrospective case series with a significant risk of bias.

Levendag et al. [20] introduced SVCI as a new RT technique, which is characterized by a smaller target volume, a reduced number of fractions, and a higher dose per fraction. In a small prospective cohort, Al-Mamgani et al. [21] showed a 2-year LC of 100% and an OS of 90% in thirty patients with T1a glottic cancer treated with SVCI. They found a comparable LC rate in comparison to patients treated at the same institution with whole-larynx irradiation. A favorable toxicity profile with few acute and no late toxicities was reported and voice quality was better preserved by SVCI. Although results after five years demonstrate a LC rate of 97.1% and an OS of 80.6% [44]. Additionally, long-term results showed an excellent laryngectomy-free survival after SVCI and improvements in the VHI score after the treatment. These results were verified by Chung et al. [45], who could demonstrate that SVCI for patients with T1a glottic tumors was feasible and lead to 3-year and 5-year LC of both 96.8% with low acute and late toxicities. Uzel et al. analyzed the outcome of patients with T1a ESGC treated with SVCI with 57.60–58.08 Gy in 15-16 fractions [46] Promising results of 18 treated patients with a LC and OS of 100% after a median follow-up of 18 months were reported. As seen in the results of Al Mamgani et al. [21], voice quality assessed by using the VHI recovered well after SVCI in this cohort (long-term follow-up limited). Based on this retrospective data, the VOCAL trial will be the first prospective study comparing vocal cord(s) only with complete laryngeal radiation in patients with T1a–b N0 glottic carcinomas [47]. It is a randomized, multicenter phase II trial with LC at 2 years being the primary endpoint.

Over the last decades, RT regimens were modified and optimized. Gowda et al. analyzed retrospectively two hundred patients with T1 glottic carcinomas treated with hypofractionated 2D-conventional RT between 1989 and 1997 [48]. Treatment of 50.0 - 52.5 Gy in 16 fractions resulted in a 5-year LC and OS of 93% and 80%, respectively. Because of the excellent LC, a low rate of severe complications, and a shorter overall treatment time, the authors concluded that this 3-week RT regimen could be beneficial. Additional publications of promising results from trials investigating hypofractionated dose regimens [21, 35, 49–52] led to explore dose-escalation with stereotactic body radiotherapy (SBRT). Schwartz et al. published feasibility and safety results from a dose-escalation phase I trial investigating three different stereotactic radiation regimens (50 Gy in 15 fractions; 45 Gy in 10 fractions; 42.5 Gy in 5 fractions) for twenty patients with cTis - T2 N0 M0 ESGC [53]. After a median follow-up of 13.4 months, they concluded that a SBRT reduction from 15 to 5 fractions was feasible without exceeding protocol-defined acute or subacute toxicity. Regarding the short follow-up, a comparable disease control to the standard treatment was observed. Results from this dose-escalating phase I trial were published by Sher et al. [54]. Local recurrences were seen in 5 of all (29) treated patients, but with no recurrence occurred in the most hypofractionated group (0/12 patients, treated with 42.5 Gy in 5 fractions). Only two patients expired dose-limiting toxicity, which were associated with large treatment volumes and active smoking status. Taken together, the authors concluded that a 5-fraction SBRT for ESGC (Tis - T2) was good tolerated and had an excellent voice quality outcome. Another phase I trial investigated SBRT with a simultaneous integrated boost to the tumor GTV in patients with cT1-2 N0 M0 glottic cancer was performed by Kang et al. [55]. Because of unexpected dose limiting toxicity in the treatment arm with 55 Gy in 11 fractions, this study was prematurely closed. Two of six patients (33%) showed grade 3 late toxicity. Another cohort in this investigation was treated with a hypofractionated dose regimen with 59.5 Gy in 17 fractions, which was well tolerated and had acceptable LC, quality of life (QOL) and voice quality. They concluded, based on the group treated with 55 Gy in 11 fractions, that SBRT is not feasible in patients with ESGC because of dose-limiting toxicity. However, maybe these conflicting results could be explained by some differences between both trials. On the one hand, Sher et al. [54] restricted the target volume to the primary tumor, whereas Kang et al. [55] treated the entire larynx with simultaneous boost to the primary tumor. On the other hand, the radiation was delivered with two different devices and techniques (robotic SBRT used by Sher et al. [54] vs VMAT used by Kang et al. [55]). We find these options require further testing and validation, because standard treatment is well tolerated with a LC already comparable to surgery.

VoiceS is comparing the time-tested organ-preserving TLM with a well-established SVCI modality head-to-head in ESGC. To our knowledge, this study is the first randomized phase III trial comparing a modern RT technique with TLM. Around one third of the required sample size is already recruited so far. Results of this study are expected to improve decision-making in the treatment of ESGC.

## Study status

Open and currently accruing since 20 November 2019. Approximate recruitment will be completed at 01 May 2025.

## Supplementary Information

**Additional file 1.** Full study protocol

**Additional file 2.** SPIRIT checklist

## Data Availability

The dataset analyzed during the current study is available from the corresponding author on reasonable request.

## Abbreviations

4D-CT: Four-dimensional computed tomography
AAA: Anisotropic analytic algorithm
AE: Adverse events
CT: Computed tomography
CTV: Clinical target volume
DVH: Dose-volume histogram
ECOG: Eastern Cooperative Oncology Group
EQ D2: Equivalent dose of 2 Gy per fraction
ESGC: Early-stage glottic cancer
GNE: Glottal-to-noise excitation ratio
GRBAS: Grade, roughness, breathiness, asthenia, strain
GTV: Gross tumor volume
HNSCC: Head and neck squamous cell carcinoma
IGRT: Image-guided radiotherapy
IMRT: Intensity-modulated radiotherapy
ITT: Intention-to-treat
JS: Jitter and Shimmer
KTP laser: Potassium-titanyl-phosphate laser
OAR: Organs at risk
PP: Per-protocol
PTV: Planning target volume
QA: Quality assurance
RBH: Roughness – breathiness – hoarseness assessment
RT: Radiotherapy
RT-QA: Radiotherapy quality assurance
SAE: Serious adverse events
SAP: Statistical analysis plan
SBRT: Stereotactic body radiotherapy
SPR: Singing power ratio
SVCI: Single vocal cord irradiation
TLM: Transoral CO_2_-laser microsurgical cordectomy
TPS: Treatment planning system
VHI: Voice handicap index
VIM: Volumetric imaging
VMAT: Volumetric modulated arc therapy
