# Supplementary material for: VoiceS: voice quality after transoral CO2 laser surgery versus single vocal cord irradiation for unilateral stage 0 and I glottic larynx cancer—a randomized phase III trial
Source: Trials. 2022 Oct 27;23:906. doi: 10.1186/s13063-022-06841-5 (PMC9615245; doi:10.1186/s13063-022-06841-5)
Supplement: Supplementary file 1 — Additional file 1. Full study protocol [file 13063_2022_6841_MOESM1_ESM.pdf]

VoiceS: Voice Quality after Transoral CO_2-Laser Surgery versus Single Vocal Cord Irradiation for Unilateral Stage 0 & I Glottic Larynx Cancer – A Randomized Phase III Trial

Clinical Study Protocol

Short Title: **VoiceS**

Study Type:	Clinical trial comparing two treatment strategies with curative intent
Study Categorization:	Risk category A according to LHR
Study Registration:	clinicaltrials.gov Number: NCT04057209<br>Cantonal Ethics Committee Number: KEK-BE 2019-01506
Sponsor-Investigator:	PD Dr. med. Olgun Elicin<br>Department of Radiation Oncology, Inselspital,<br>Bern University Hospital and University of Bern, Bern, Switzerland.<br>Phone: +41 31 632 24 31<br>Fax: +41 31 632 85 67<br>E-mail: olgun.elicin@insel.ch
Investigational Treatments:	1) Transoral CO_2-Laser Microsurgical Cordectomy<br>2) Single Vocal Cord Irradiation
Protocol Version and Date:	1.4 – 01.12.2021

Change history

Version Number	Version date	Modified without version change	Description, comments	Control
1.0	10.07.2019		Initial version	OE
1.1	09.09.2019	no	Amended upon the request of the ethics committees	OE
1.2	30.10.2019	no	Amended upon the request of the ethics committees	OE
1.3	20.11.2019	no	The sketch in section 8.1 was changed. More details in definitions of SAE added upon the request of the Clinical Trials Unit, Bern	OE
1.2	01.12.2021	no	non-substantial amendment: minor typo corrections, clarifications, minor technical modifications for surgical and radiotherapy techniques and QA procedures	OE

CONFIDENTIAL

The information contained in this document is confidential and the property of the sponsor investigator. The information may not - in full or in part - be transmitted, reproduced, published, or disclosed to others than the applicable competent ethics committees and regulatory authorities without prior written authorization from the sponsor except to the extent necessary to obtain informed consent from those who will participate in the study.

Signature Pages

Study Title VoiceS: Voice Quality after Transoral CO_2-Laser Surgery versus Single Vocal Cord Irradiation for Unilateral Stage0 & I Glottic Larynx Cancer – A Randomized Phase III Trial

The Sponsor-Investigator and trial statistician have approved the protocol version 1.4 (01.12.2021), and confirm hereby to conduct the study according to the protocol, current version of the World Medical Association Declaration of Helsinki, ICH-GCP guidelines or ISO 14155 norm if applicable and the local legally applicable requirements.

Sponsor-Investigator:

Olgun Elicin

Bern, 01.12.2021	Olgun Elicin
Place/Date	Signature

Local Principal Investigators at study sites:

I have read and understood this trial protocol and agree to conduct the trial as set out in this study protocol, the current version of the World Medical Association Declaration of Helsinki, ICH-GCP guidelines and the local legally applicable requirements.

Site ____

____

Principal investigator ____

________________________ ________________________________

Place/Date Signature

Table of Contents

STUDY SYNOPSIS **7**
STUDY SUMMARY IN LOCAL LANGUAGE **13**
ABBREVIATIONS **14**
STUDY SCHEDULE **16**
1. STUDY ADMINISTRATIVE STRUCTURE **17**
1.1 Sponsor-Investigator 17
1.2 Co-Investigators 17
1.3 Local Principal Investigators 19
1.3.1 University Hospital of Geneva 19
1.3.2 University Hospital of Lausanne 20
1.3.3 University Hospital of Zurich 20
1.4 Statistician ("Biostatistician") 20
2. ETHICAL AND REGULATORY ASPECTS **21**
2.1 Study registration 21
2.2 Categorization of study 21
2.3 Competent Ethics Committee (CEC) 21
2.4 Competent Authorities (CA) 21
2.5 Ethical Conduct of the Study 21
2.6 Declaration of interest 21
2.7 Patient Information and Informed Consent 21
2.8 Participant privacy and confidentiality 22
2.9 Early termination of the study 22
2.10 Protocol amendments 22
3. BACKGROUND AND RATIONALE **23**
3.1 Background and Rationale 23
3.2 Investigational Treatments and Indication 23
3.3 Clinical Evidence to Date 23
3.4 Risks / Benefits 24
3.5 Justification of Choice of Study Population 24
4. STUDY OBJECTIVES **25**
4.1 Overall Objective 25
4.2 Primary Objective 25
4.3 Secondary Objectives 25
4.4 Safety Objectives 25
5. STUDY OUTCOMES **25**
5.1 Primary Outcome 25
5.2 Secondary Outcomes 25
5.3 Other Outcomes of Interest 25
5.4 Safety Outcomes 25
6. STUDY DESIGN **26**
6.1 General study design and justification of design 26
6.2 Methods of minimizing bias 26
6.2.1 Randomization 26
6.2.2 Blinding procedures 26

7. STUDY POPULATION ... 27
7.1 Inclusion criteria ... 27
7.2 Exclusion criteria ... 27
7.3 Recruitment and screening ... 27
7.4 Assignment to study groups and randomization ... 28
7.5 Criteria for withdrawal / discontinuation of participants ... 28
8. STUDY INTERVENTIONS ... 29
8.1 Identity of Study Interventions ... 29
8.2 Transoral CO_2-Laser Microsurgical Cordectomy (TLM) in Arm A ... 30
8.2.1 Preoperative Assessment ... 30
8.2.2 Definition of Technique and Resection Margins ... 30
8.2.3 Post-surgical Management of Close or Positive Margins ... 31
8.2.4 Post-operative Care ... 31
8.2.5 Expected Complications / Surgical Adverse Events of TLM ... 31
8.2.6 Standardization of the Surgical Technique ... 31
8.2.7 Quality Assurance (QA) Procedures (Adequacy of the Oncologic Surgery) ... 32
8.3 Single Vocal Cord Irradiation (SVCI) in Arm B ... 34
8.3.1 Equipment and Tools ... 34
8.3.2 General Considerations for Treatment Planning ... 34
8.3.3 Patient Positioning ... 34
8.3.4 Simulation (Patient Image Acquisition) ... 34
8.3.5 Definition of Volumes ... 34
8.3.6 Treatment Technique, Treatment Planning and Dose Prescription ... 35
8.3.7 Treatment Delivery and Verification with Online Imaging ... 37
8.3.8 Fractionation Schedule ... 38
8.3.9 Facility Questionnaire and Dosimetry Audit ... 38
8.3.10 Radiotherapy Quality Assurance (RT-QA) ... 38
8.3.11 Dummy Runs ... 38
8.4 Compliance with study intervention ... 39
8.5 Data Collection and Follow-up for withdrawn participants ... 39
8.6 Trial specific preventive measures ... 39
8.7 Concomitant Interventions (treatments) ... 39
9. STUDY ASSESSMENTS ... 40
9.1 Study flow charts ... 40
9.2 Assessments of outcomes ... 40
9.2.1 Assessment of primary outcome ... 41
9.2.2 Assessment of secondary outcomes ... 41
9.2.3 Assessment of safety outcomes ... 41
9.2.4 Assessments in participants who prematurely stop the study ... 42
9.3 Procedures at each visit ... 42
10. SAFETY ... 42
10.1 Exceptions ... 42
10.2 Severity assessment ... 43
10.3 Causality assessment ... 43
10.4 Expectedness assessment ... 43

10.5 SAE Reporting Procedure ... 43
10.6 Reporting to the Ethics Committee ... 44
10.7 Reporting and Handling of Pregnancies ... 44
10.8 Periodic reporting of safety ... 44
11. STATISTICAL METHODS ... 45
11.1 Hypothesis and Determination of Sample Size ... 45
11.2 Randomization and Stratification ... 45
11.3 Planned Analyses ... 45
11.3.1 Datasets to be analyzed, analysis populations ... 45
11.3.2 Primary Analysis ... 45
11.3.3 Secondary Analyses ... 46
11.3.4 Deviation(s) from the original statistical plan ... 46
11.4 Handling of missing data and drop-outs ... 46
12. QUALITY ASSURANCE AND CONTROL ... 47
12.1 Data handling and record keeping / archiving ... 47
12.1.1 Case Report Forms ... 47
12.1.2 Specification of source documents ... 47
12.1.3 Record keeping / archiving ... 47
12.2 Data management ... 47
12.2.1 Data Management System ... 47
12.2.2 Data security, access and back-up ... 47
12.2.3 Data transfer for radiotherapy data and sound recordings ... 48
12.2.4 Analysis and archiving ... 48
12.2.5 Electronic and central data validation ... 48
12.3 Monitoring ... 48
12.4 Audits and Inspections ... 48
12.5 Confidentiality, Data Protection ... 48
13. PUBLICATION AND DISSEMINATION POLICY ... 49
14. FUNDING AND SUPPORT ... 49
14.1 Funding ... 49
14.2 Other Support ... 49
15. INSURANCE ... 49
16. REFERENCES ... 50
17. APPENDICES ... 53

STUDY SYNOPSIS

**Sponsor / Sponsor-Investigator**	Olgun Elicin
**Study Title:**	VoiceS: Voice Quality after Transoral CO_2-Laser Surgery versus Single Vocal Cord Irradiation for Unilateral Stage 0 & I Glottic Larynx Cancer – A Randomized Phase III Trial
**Short Title**	VoiceS
**Protocol Version and Date:**	1.4 – 01.12.2021
**Trial registration:**	clinicaltrials.gov; Swiss National Clinical Trials Portal
**Study category and Rationale**	Category A: the treatments involved in the study are not experimental and being used as two distinct standards of care.
**Clinical Phase:**	phase III
**Background and Rationale:**	Laser surgery and radiotherapy are well-established standards of care for unilateral stage 0 & I carcinoma in situ (Cais) and squamous cell carcinoma of glottic larynx (SCCGL). Based on meta-analyses, functional and oncological outcome after both treatment modalities are comparable[1–5]. However, no properly conducted randomized trials comparing these treatments exist. The only such trial with the endpoint of voice quality had to be prematurely closed due to low accrual[6].<br><br>The traditional radiotherapy involves the treatment of the whole larynx. Recently, a new radiotherapy technique was introduced by a team of researchers from Netherlands, where the treated target volume consists of involved vocal cord and therefore 8 to 10-fold smaller than the target volumes used for traditional whole larynx irradiation. The treatment is reduced to 16 fractions which corresponds to 3 weeks and a day[7–12]. The results of a prospective cohort (n=30) with single vocal cord irradiation (SVCI) were compared with the results of a historical prospective cohort previously treated with whole larynx radiotherapy (n=131) in the same institute. The median follow-up was 30 months. The voice handicap index (VHI) at all time points beginning from the 6th week after SVCI was significantly superior to the same time points with conventional radiotherapy. Moreover, a comparable local control with SVCI (100%) vs. conventional radiotherapy (92%) was reported at two years, p=0.24[12]. Based on that, the SVCI technique was implemented as a standard approach for SCCGL in many radiation oncology clinics in the recent years.<br><br>Based on this information, our main aim is to compare SVCI to Transoral CO_2-Laser Microsurgical Cordectomy (TLM) with the main focus of patient-reported voice quality.
**Objective(s):**	Primary objective: Assessment of the patient-reported subjective voice quality between 6 and 24 months after treatment.<br><br>Secondary objectives:<br><br>- Qualitative post-treatment comparison of the voice in two study arms<br><br>- Quantitative post-treatment comparison of the voice in two study arms<br><br>- Comparison of the oncological outcome between study arms.<br><br>- Assessment of late toxicity among study arms.
**Outcome(s):**	Primary endpoint: average of the VHI at 6, 12, 18, and 24 months<br><br>Secondary endpoints:

	- Perceptual impression of the voice via Roughness – Breathiness - Hoarseness (RBH) assessment at the above-mentioned time points.<br>- Quantitative characteristics of voice by means of Jitter and Shimmer (JS), Glottal-to-Noise Excitation Ratio (GNE) and Singing Power Ratio (SPR), which will be assessed at the above-mentioned time points<br>- Loco-regional control of the disease at 2 and 5 years<br>- Treatment toxicity at 2 and 5 years based on CTCAE v.5.0
**Study design:**	Multicenter randomized phase III, non-blinded (blinding not possible due to the obvious differences between both treatments)
**Inclusion criteria:**	1. ECOG performance status 0-1 at the time of registration<br>2. ≥18 years of age<br>3. Baseline assessments and documentation of voice quality by means of VHI, JS, RBH, GNE, SPR<br>4. Histopathologically confirmed, previously untreated unilateral (cT1a or unilateral cTis) stage 0 or I glottic larynx cancer based on the UICC staging system (8th edition).<br>5. History and physical examination by treating physician (head and neck surgeon and radiation oncologist) within 28 days prior registration<br>6. The patient must be expected to withstand both study interventions<br>7. The patient must have undergone panendoscopy with assessment for the feasibility of transoral exposure for resection. Patients without feasible exposure are not eligible<br>8. Localization of the tumor should allow resection with a minimum of 2 mm macroscopical margin without extension to the contralateral vocal fold, without partial resection of the arytenoid cartilage and without resection of parts of thyroid cartilage (Cordectomy Type I-IV according the classification of the European Laryngological Society)[13]<br>9. Hemoglobin ≥10 g/dL or 6.2 mmol/L (Note: The use of transfusion to achieve Hgb ≥10 g/dL is acceptable) within the 28 days prior to accrual<br>10. Women with child-bearing potential and using effective contraception, and not pregnant and agree not to become pregnant (see section 8.6) during participation in the trial and 30 days after radiotherapy. A negative pregnancy test before inclusion (within 28 days) into the trial is required for all women with child-bearing potential. Men agree not to father a child during participation in the trial and 30 days after radiotherapy<br>11. Written informed consent, signed by the patient and the investigator
**Exclusion Criteria**	1. Infection hampering the voice quality at the time of voice assessment<br>2. Involvement of the anterior commissure by the tumor<br>3. Previous oncologic surgery with curative intent (exception: excisional biopsies resulting in unacceptable close R0 or R1/R2 margins may be included) or radiotherapy to larynx<br>4. Synchronous or previous malignancies. Exceptions are adequately treated basal cell carcinoma or SCC of the skin, or in situ carcinoma of the cervix uteri, low-risk prostate cancer or breast with a cancer-free follow-up time of at least 3 years, or other previous malignancy with a progression-free interval of at least 5 years.<br>5. Co-existing disease prejudicing survival (expected survival less than 6 months).<br>6. Active bacterial or fungal infection requiring intravenous antibiotics at the time of registration<br>7. History of any voice disorders (not related to the SCCGL) lasting longer than 3 weeks<br>8. Illness requiring hospitalization or precluding study therapy within 28 days before registration<br>9. Presence of any psychological, familial, sociological or geographical

	condition potentially hampering compliance with the study protocol and follow-up schedule; those conditions should be discussed with the patient before registration in the trial
**Measurements and procedures:**	1) Baseline assessments of voice parameters as defined under endpoints<br>2) Treatment based on randomization<br>3) Follow-up per protocol
**Study Intervention:**	Arm B: Single Vocal Cord Irradiation (SVCI)
**Control Intervention:**	Arm A: Transoral CO_2-Laser Microsurgical Cordectomy (TLM)
**Number of Participants with Rationale:**	The sample size calculation is based on the primary outcome, the VHI at 6 to 24 months (averaged). The working hypothesis is that there is a difference between the two intervention groups in regard to VHI. Al-Mamgani et al. (2015) reported standard deviations for VHI ranging from 2 to 10 score points. A difference of 8 points between the two groups are regarded as clinically relevant and a conservative standard deviation of 8 points is assumed. Based on a two-sample means test, 34 patients (17 per group) are needed to detect a difference in VHI at a two-sided alpha-level of 0.05 with a power of 80%.<br>This sample size calculation is conservative. In the analysis, the average difference over four time points (6, 12, 18, and 24 months) will be modelled and additionally adjusted for the baseline VHI to yield more power.
**Study Duration:**	48 months for primary and secondary endpoints, 84 months for late secondary endpoints
**Study Schedule:**	*: Ethics approval and registration of the study, start of patient screening and accrual<br>* + 24 months: last patient last treatment<br>* + 48 months: last patient last visit for the primary endpoint<br>* + 51 months: statistical analysis, preparation of the manuscript and submission of the manuscript to a peer-reviewed scientific journal regarding the primary endpoint and the secondary endpoints with 2-years follow-up<br>* + 84 months: last patient last visit for final long-term outcomes<br>* + 87 months: statistical analysis, preparation of the manuscript and submission of the manuscript to a peer-reviewed scientific journal regarding the long-term oncologic and toxicity endpoints with 5-years follow-up.
**Investigator(s):**	**Sponsor-Investigator:**<br>Olgun Elicin<br>Department of Radiation Oncology<br>Bern University Hospital and University of Bern<br>Inselspital, Freiburgstrasse, 3010 Bern<br>Phone: +41 31 632 24 31<br>Fax: +41 31 632 85 67<br>E-mail: olgun.elicin@insel.ch<br><br>**Co-Investigators:**<br>Roland Giger<br>Department of Oto-Rhino-Laryngology, Head and Neck Surgery<br>Bern University Hospital and University of Bern<br>Inselspital, Freiburgstrasse, 3010 Bern

	Phone: +41 31 632 96 33<br>Fax: +41 31 632 88 09<br>E-mail: roland.giger@insel.ch<br><br>Eberhard Seifert<br>Division of Phoniatrics, Department of Otorhinolaryngology, Head and Neck Surgery<br>Inselspital, Freiburgstrasse, 3010 Bern<br>3010 Bern<br>Phone: +41 31 632 33 49<br>Fax: +41 31 632 31 93<br>E-mail: eberhard.seifert@insel.ch<br><br>Abrahim Al-Mamgani<br>Department of Radiation Oncology<br>Netherlands Cancer Institute/Antoni van Leeuwenhoek,<br>1066 CX Amsterdam<br>Phone: +31 20 512 21 35<br>Fax: +31 20 669 11 01<br>E-mail: a.almamgani@nki.nl<br><br>Mohamed Shelan<br>Department of Radiation Oncology<br>Bern University Hospital and University of Bern<br>Inselspital, Freiburgstrasse, 3010 Bern<br>Phone: +41 31 632 24 31<br>Fax: +41 31 632 85 67<br>E-mail: mohamed.shelan@insel.ch<br><br>Elena Riggenbach<br>Department of Radiation Oncology<br>Bern University Hospital and University of Bern<br>Inselspital, Freiburgstrasse, 3010 Bern<br>Phone: +41 31 632 24 31<br>Fax: +41 31 632 85 67<br>E-mail: elena.riggenbach@insel.ch<br><br>Dario Terribilini<br>Department of Radiation Oncology, Division of Medical Radiation Physics, Bern University Hospital and University of Bern,<br>Inselspital, Freiburgstrasse, 3010 Bern<br>Phone: +41 31 632 18 98<br>Fax: +41 31 632 26 76<br>E-mail: dario.terribilini@insel.ch

	Andreas Joosten<br>Department of Radiation Oncology, Division of Medical Radiation Physics, Bern University Hospital and University of Bern,<br>Inselspital, Freiburgstrasse, 3010 Bern<br>Phone: +41 31 632 18 98<br>Fax: +41 31 632 26 76<br>E-mail: andreas.joosten@insel.ch<br><br>Peter Manser<br>Department of Radiation Oncology, Division of Medical Radiation Physics, Bern University Hospital and University of Bern,<br>Inselspital, Freiburgstrasse, 3010 Bern<br>Phone: +41 31 632 18 98<br>Fax: +41 31 632 26 76<br>E-mail: peter.manser@insel.ch<br><br>Matthias Dettmer<br>Department of Pathology, University of Bern<br>Murtenstrasse 31, 3010 Bern<br>Phone: +41 31 632 99 69<br>Fax: +41 31 632 49 95<br>E-mail: matthias.dettmer@pathology.unibe.ch<br><br>Christian Simon<br>Department of Otolaryngology - Head and Neck Surgery,<br>CHUV University of Lausanne<br>Rue du Bugnon, 2, 1011 Lausanne<br>Phone: +41 213140539<br>Fax: +41 213142706<br>e-mail: christian.simon@chuv.ch<br><br>E. Mahmut Ozsahin<br>Department of Radiation Oncology, Centre Hospitalier Universitaire Vaudois, 1011 Lausanne<br>Phone: +41 21 314 46 03<br>Fax: +41 21 314 46 01<br>E-mail: esat-mahmut.ozsahin@chuv.ch<br><br>Raphaël Moeckli<br>Institut of Radiation Physics,<br>Rue du Grand-Pré 1, 1007 Lausanne<br>Phone: +41 21 314 46 18<br>Fax: +41 21 314 82 99<br>E-mail: raphael.moeckli@chuv.ch

	Andreas Limacher<br>CTU Bern, University of Bern<br>Mittelstrasse 43, 3012 Bern<br>Phone: +41 31 631 35 10<br>e-mail: andreas.limacher@ctu.unibe.ch<br><br>Francesca Caparrotti<br>Department of Radiation Oncology, Genève University Hospital,<br>Rue Gabrielle-Perret-Gentil 4,<br>1205 Genève<br>Phone: +41 79 553 56 12<br>Fax: +41 22 372 71 17<br>E-mail: francesca.caparrotti@hcuge.ch<br><br>Jean Bourhis<br>Department of Radiation Oncology, CHUV,<br>University of Lausanne<br>Rue du Bugnon, 21<br>1011 Lausanne<br>Phone: +41 21 314 46 66<br>Fax: +41 21 314 02 00<br>E-mail: jean.bourhis@chuv.ch<br><br>PD. Dr. Martina Broglie Däppen<br>Department of Head and Neck Surgery, University Hospital of Zurich<br>Rämistrasse 100, 8091 Zürich<br>Phone: +41 (0) 44 225 15 36<br>Fax: N/A<br>E-mail: martina.brogliedaeppen@usz.ch
**Study Centre(s):**	Multi-center study.<br>Number of projected centers: 3<br>- Bern University Hospital and University of Bern, Inselspital, Freiburgstrasse, 3010 Bern, Switzerland<br>- Centre Hospitalier Universitaire Vaudois, 1011 Lausanne, Switzerland<br>- Geneve University Hospital, 1205 Geneva, Switzerland
**Statistical Considerations:**	Please refer to the section “Number of Participants with Rationale” above.
**GCP Statement:**	This study will be conducted in compliance with the protocol, the current version of the Declaration of Helsinki, the ICH-GCP or ISO EN 14155 (as far as applicable) as well as all national legal and regulatory requirements.

STUDY SUMMARY IN LOCAL LANGUAGE

– German –

Für Stimmlippenkarzinome im Frühstadium bestehen zwei gleichwertige Therapiemöglichkeiten: endoskopische Laserchirurgie oder Strahlentherapie. Gemäss medizinischer Literatur zeigen beide Behandlungsmodalitäten vergleichbare Resultate bezüglich Heilungschancen der Tumorerkrankung und Stimmqualität. Allerdings sind alle bisher durchgeführten und publizierten Studien mit nicht idealer Methodologie durchgeführt worden. Patienten, die in diesen Studien bewertet wurden, haben sich entweder selber aktiv für eine der beiden Therapiearten entschieden oder ihre ‚richtige' Behandlung wurde ihnen von den behandelnden Ärzten vorgegeben'.

Unsere Studie legt den Hauptfokus auf die langfristige Stimmqualität. Im Rahmen dieser Studie wird die Therapieart eines/-r jeden/-r Patienten/-in (Chirugie oder Radiotherapie) nicht von den Ärzten oder den Patienten/-innen selber, sondern randomisiert mit einem Computerprogramm (Zufallsgenerator) bestimmt. Dadurch wird es möglich sein, Evidenz von höchster wissenschaftlicher Qualität zu generieren. Die chirurgischen und radiotherapeutischen Behandlungen werden heutzutage mit modernsten organschonenden Techniken durchgeführt, und bezüglich Qualität regelmässig überprüft.

Die vorab durchgeführten biostatistischen Berechnungen zeigen, dass 34 Patienten/-innen benötigt werden, um die Studie erfolgreich und mit einer genügend hohen statistischen Aussagekraft abzuschliessen. In jedem Kanton, in dem die Studie durchgeführt wird, erfolgt eine Überprüfung durch die jeweils zuständige Ethikkommission.

– French –

Pour un carcinome précoce de la corde vocale, deux types de traitements avec une efficacité similaire sont possibles : la chirurgie endoscopique au laser CO_2 ou la radiothérapie exclusive. Selon la littérature médicale, les deux stratégies thérapeutiques montrent des résultats similaires concernant le pronostic et la qualité de la voix. Néanmoins, la méthodologie de toutes les études effectuées et publiées n'était pas idéale. Les patients évalués par ces études ont soit choisi eux-mêmes le type de traitement ou ont suivi l'avis thérapeutique du médecin spécialisé.

Le but principal de notre étude est l'évaluation de la qualité de la voix à long terme. Dans le cadre de cette étude, le choix du type de traitement (chirurgie endoscopique au laser CO_2 ou radiothérapie exclusive) pour chaque patient/-e sera décidé par randomisation informatisée (au hasard) et non pas par le/la patient/-e ou le médecin. Par cette méthode, il sera possible de générer des résultats scientifiques de haute qualité.

Les techniques modernes des traitements chirurgicaux et radiothérapeutiques d'aujourd'hui ont pour but de préserver le plus possible les tissus sains. Elles subissent de réguliers contrôles de qualité.

Les calculs biostatistiques déjà effectués montrent que 34 patients/-es sont nécessaires pour terminer l'étude avec succès et obtenir des résultats statistiquement significatifs.

Dans chaque canton, dans lequel l'étude sera effectuée, la commission d'éthique concernée examinera le protocole et le déroulement de celle-ci.

ABBREVIATIONS

AE	Adverse Event
CA	Competent Authority (e.g. Swissmedic)
CEC	Competent Ethics Committee
CRF	Case Report Form
ClinO	Ordinance on Clinical Trials in Human Research *(in German: KlinV, in French: OClin)*
eCRF	Electronic Case Report Form
CTCAE	Common terminology criteria for adverse events
DSUR	Development safety update report
GCP	Good Clinical Practice
GNE	Glottal-to-Noise Excitation Ratio
IB	Investigator's Brochure
Ho	Null hypothesis
JS	Jitter and Shimmer
H1	Alternative hypothesis
HFG	Humanforschungsgesetz (Law on human research)
HMG	Heilmittelgesetz
HRA	Federal Act on Research involving Human Beings
IMP	Investigational Medicinal Product
IIT	Investigator-initiated Trial
ISO	International Organisation for Standardisation
ITT	Intention to treat
KlinV	Verordnung über klinische Versuche in der Humanforschung *(in English: ClinO, in French OClin)*
LPTh	Loi sur les produits thérapeutiques
LRH	Loi fédérale relative à la recherche sur l'être humain
MD	Medical Device
OClin	Ordonnance sur les essais cliniques dans le cadre de la recherche sur l'être humain *(in German : KlinV, in English : ClinO)*
PI	Principal Investigator
QA	Quality Assurance
RBH	Roughness – Breathiness – Hoarseness
RT	Radiotherapy
RT-QA	Radiotherapy Quality Assurance
SDV	Source Data Verification
SCCGL	Squamous cell carcinoma of glottic larynx (for convenience, both carcinoma in situ and squamous cell carcinoma of glottic larynx will be mentioned as SCCGL throughout the protocol)
SOP	Standard Operating Procedure

SPC	Summary of product characteristics
SPR	Singing Power Ratio
SVCI	Single vocal cord irradiation
TLM	Transoral CO_2-Laser Microsurgical Cordectomy
TMF	Trial Master File
UAR	Unexpected Adverse Reaction
UICC	Union for International Cancer Control
VIM	volumetric imaging
VHI	Voice handicap index

STUDY SCHEDULE

Table 1: Summary flowchart

Study Periods ➔	Screening and Enrollment	Treatment Period	Follow-up until 60 months <u>after enrollment</u>
Time unit ➔	Days	Weeks	Months
Patient Information and Informed Consent	-28 – 0		
Medical history and demographics	-28 – 0		
Panendoscopy, assessment of exposure and staging according to institutional local standards	-28 – 0		
Eligibility Criteria Check	-28 – 0		
SCCGL-oriented physical examination	-28 – 0		
VHI[#], RBH, GNE, SPR, JS	-28 – 0		(1.5)[#], 6, 12, 18, 24 (+/-15 days)
Enrollment and randomization*	0		
Endoscopic assessment of the index tumor, symptom oriented physical examination	-28 – 0		every 3 months (+/-15 days) until 24th month<br>every 6 months (+/-15 days) between 24th and 60th months
Evaluation of actual smoking status and toxicity according to CTCAE v.5.0 (Appendix A)	-28 – 0 (baseline smoking status and toxicity[¥])	weekly during radiotherapy and within one week of surgery: no need to document except for SAEs	
Pregnancy test for women with child-bearing potential	-28 – 0	3 – 7[Ψ]	until 3rd month[Ψ]
Arm A: Transoral CO_2-Laser Microsurgical Cordectomy[∇]		day of surgery +/- day of re-resection	
Arm B: Single Vocal Cord Irradiation[∇]		3 (16 fractions)	

GNE: Glottal-to-Noise Excitation Ratio; JS: Jitter and Shimmer; RBH: Roughness – Breathiness – Hoarseness; SAEs: serious adverse events; SCCGL: squamous cell carcinoma of glottic larynx; SPR: Singing Power Ratio; VHI: voice handicap index

*: Even if done on the same day, patient enrollment shall be done after the completion of the necessary documentation

#: VHI at 6 weeks after the end of treatment (regardless of study arm) will be assessed to evaluate the need for phoniatric rehabilitation, but <u>not</u> as a study-specific outcome measure (i.e. primary endpoint) to avoid lead time bias. Patients with a VHI > 14 will be recommended to undergo a phoniatric rehabilitation, but <u>the examiner should not try to persuade the patient</u>. Similarly, <u>any VHI >14 at any follow-up time point</u> or <u>patients' request</u> justifies the need for phoniatric rehabilitation, which will be recommended to the patients regardless of study arm. No need to record the VHI in the CRFs.

¥: The baseline toxicity scores and smoking status may be documented after the accrual but it must be done before the start of trial treatment.

Ψ: If clinically indicated

∇: The treatment shall start within 28 days post-accrual

1. STUDY ADMINISTRATIVE STRUCTURE

1.1 Sponsor-Investigator

Olgun Elicin
Department of Radiation Oncology
Bern University Hospital and University of Bern
Inselspital
Freiburgstrasse
3010 Bern
Phone: +41 31 632 24 31
Fax: +41 31 632 85 67
E-mail: olgun.elicin@insel.ch

The Sponsor-Investigator leads the research team in the establishment of the trial design. He is responsible for the establishment of the main documents (Protocol, CRF, patient information, consent form, and insurance documents), grant applications to secure funding, as well as applications to the CECs. During the trial, he delegates investigators as necessary and ensures proper documentation.

1.2 Co-Investigators

Roland Giger
Department of Oto-Rhino-Laryngology, Head and Neck Surgery
Bern University Hospital and University of Bern
Inselspital
Freiburgstrasse
3010 Bern
Phone: +41 31 632 96 33
Fax: +41 31 632 88 09
E-mail: roland.giger@insel.ch

Responsible for the quality assurance and conduction of surgery

Eberhard Seifert
Division of Phoniatrics, Department of Otorhinolaryngology, Head and Neck Surgery
Inselspital, Freiburgstrasse, 3010 Bern
3010 Bern
Phone: +41 31 632 33 48
Fax: +41 31 632 88 09
E-mail: eberhard.seifert@insel.ch

Responsible for the quality and conduction of voice quality-related assessments

Abrahim Al-Mamgani
Department of Radiation Oncology
Netherlands Cancer Institute/Antoni van Leeuwenhoek,
1066 CX Amsterdam
Phone: +31 20 512 21 35
Fax: +31 20 669 11 01
E-mail: a.almamgani@nki.nl

Responsible for the establishment of the radiotherapy concept and protocol development

Mohamed Shelan
Department of Radiation Oncology
Bern University Hospital and University of Bern
Inselspital, Freiburgstrasse, 3010 Bern
Phone: +41 31 632 24 31
Fax: +41 31 632 85 67
E-mail: mohamed.shelan@insel.ch

Responsible for the quality and establishment of study-specific documents

Elena Riggenbach
Department of Radiation Oncology
Bern University Hospital and University of Bern
Inselspital, Freiburgstrasse, 3010 Bern
Phone: +41 31 632 24 31
Fax: +41 31 632 85 67
E-mail: elena.riggenbach@insel.ch

Responsible for the quality and establishment of study-specific documents

Dario Terribilini
Department of Radiation Oncology, Division of Medical Radiation Physics, Bern University Hospital and University of Bern,
Inselspital, Freiburgstrasse, 3010 Bern
Phone: +41 31 632 18 98
Fax: +41 31 632 26 76
E-mail: dario.terribilini@insel.ch

Responsible for the radiotherapy planning and quality assurance

Andreas Joosten
Department of Radiation Oncology, Division of Medical Radiation Physics, Bern University Hospital and University of Bern,
Inselspital, Freiburgstrasse, 3010 Bern
Phone: +41 31 632 18 98
Fax: +41 31 632 26 76
E-mail: andreas.joosten@insel.ch

Responsible for the radiotherapy planning and quality assurance

Peter Manser
Department of Radiation Oncology, Division of Medical Radiation Physics, Bern University Hospital and University of Bern,
Inselspital, Freiburgstrasse, 3010 Bern
Phone: +41 31 632 18 98
Fax: +41 31 632 26 76
E-mail: peter.manser@insel.ch

Responsible for the radiotherapy planning and quality assurance

Matthias Dettmer
Department of Pathology, University of Bern
Murtenstrasse 31, 3010 Bern
Phone: +41 31 632 99 69
Fax: +41 31 632 49 95
E-mail: matthias.dettmer@pathology.unibe.ch

Responsible for central pathology review and quality assurance

Christian Simon
Department of Otolaryngology - Head and Neck Surgery, CHUV
University of Lausanne
Rue du Bugnon, 21
1011 Lausanne
Phone: +41 213140539
Fax: +41 213142706
e-mail: christian.simon@chuv.ch

Responsible for the quality and conduction of surgery

E. Mahmut Ozsahin
Department of Radiation Oncology, CHUV,
University of Lausanne
Rue du Bugnon, 21
1011 Lausanne
Phone: +41 21 314 46 03
Fax: +41 21 314 46 01
E-mail: esat-mahmut.ozsahin@chuv.ch

Responsible for the quality and conduction of radiotherapy

Raphaël Moeckli
Institut of Radiation Physics, Rue du Grand-Pré 1
1007 Lausanne
Phone: +41 21 314 46 18
Fax: +41 21 314 82 99
E-mail: raphael.moeckli@chuv.ch

Responsible for the radiotherapy planning and quality assurance

1.3 Local Principal Investigators

1.3.1 University Hospital of Geneva

Francesca Caparrotti
Department of Radiation Oncology, Genève University Hospital,
Rue Gabrielle-Perret-Gentil 4,
1205 Genève
Phone: +41 79 553 56 12
Fax: +41 22 372 71 17
E-mail: francesca.caparrotti@hcuge.ch

1.3.2 University Hospital of Lausanne

Jean Bourhis
Department of Radiation Oncology, CHUV,
University of Lausanne
Rue du Bugnon, 21
1011 Lausanne
Phone: +41 21 314 46 66
Fax: +41 21 314 02 00
E-mail: jean.bourhis@chuv.ch

1.3.3 University Hospital of Zurich

PD. Dr. Martina Broglie Däppen
Department of Head and Neck Surgery, University Hospital of Zurich
Rämistrasse 100, 8091 Zürich
Phone: +41 (0) 44 225 15 36
Fax: N/A
E-mail: martina.brogliedaeppen@usz.ch

1.4 Statistician ("Biostatistician")

Andreas Limacher
CTU Bern, University of Bern
Mittelstrasse 43
3012 Bern
Phone: +41 31 631 35 10
e-mail: andreas.limacher@ctu.unibe.ch

2. ETHICAL AND REGULATORY ASPECTS

The decision of the CEC concerning the conduct of the study will be made in writing to the Sponsor-Investigator before commencement of this study. The clinical study can only begin once approval from all required authorities has been received. Any additional requirements imposed by the authorities shall be implemented.

2.1 Study registration

The study will be listed in clinicaltrials.gov and in the Swiss National Clinical Trials Portal.

2.2 Categorization of study

Category A: the treatments involved in the study are not experimental and being used as two distinct standards of care.

2.3 Competent Ethics Committee (CEC)

The responsible investigator at each site ensures that approval from an appropriately constituted CEC is sought for the clinical study.

Approval from all involved CECs is required prior to the start of the study. No changes to the protocol will be made without prior approval from the CECs, except if necessary to eliminate immediate hazards to participants. Premature study end or interruption of the study is reported within 15 days. The regular end of the study is reported to the lead CEC within 90 days, the final study report shall be submitted within one year after study end. Amendments are reported according to chapter 2.10.

2.4 Competent Authorities (CA)

The regular end of the study is reported to the CEC within 90 days, the final study report shall be submitted within one year after study end. Amendments are reported according to chapter 2.10, except for non-substantial amendments that are reported as soon as possible.

2.5 Ethical Conduct of the Study

The study will be carried out in accordance to the protocol and with principles enunciated in the current version of the Declaration of Helsinki, the guidelines of Good Clinical Practice (GCP) issued by ICH, the Swiss Law and Swiss regulatory authority's requirements. The CEC and regulatory authorities will receive annual safety and interim reports and be informed about study stop/end in agreement with local requirements.

2.6 Declaration of interest

The investigators declare that they have no conflict of interest.

2.7 Patient Information and Informed Consent

The investigators will explain to each participant the nature of the study, its purpose, the procedures involved, the expected duration, the potential risks and benefits and any discomfort it may entail. Each participant will be informed that the participation in the study is voluntary and that he/she may withdraw from the study at any time and that withdrawal of consent will not affect his/her subsequent medical assistance and treatment.

The participant will be informed that his/her medical records may be examined by authorized individuals other than their treating physician.

All participants for the study will be provided a participant information sheet and a consent form describing the study and providing sufficient information for participant to make an informed decision about their participation in the study.

The patient information sheet and the consent form will be submitted to the CEC to be reviewed and approved. The formal consent of a participant, using the approved consent form, must be obtained before the participant is submitted to any study procedure.

The participant should read and consider the statement for at least 24 hours before signing and dating the informed consent form and should be given a copy of the signed document. The consent form must

also be signed and dated by the investigator (or his designee) and it will be retained as part of the study records.

2.8 Participant privacy and confidentiality

The investigators affirm and uphold the principle of the participant's right to privacy and that they shall comply with applicable privacy laws. Especially, anonymity of the participants shall be guaranteed when presenting the data at scientific meetings or publishing them in scientific journals.

Individual subject medical information obtained as a result of this study is considered confidential and disclosure to third parties is prohibited. Subject confidentiality will be further ensured by utilizing subject identification code numbers to correspond to treatment data in the computer files.

For data verification purposes, authorized representatives a competent authority or the CEC may require direct access to parts of the medical records relevant to the study, including participants' medical history.

2.9 Early termination of the study

The Sponsor-Investigator may terminate the study prematurely according to certain circumstances, for example:

- ethical concerns,
- insufficient participant recruitment,
- when the safety of the participants is doubtful or at risk, respectively,
- alterations in accepted clinical practice that make the continuation of a clinical trial unwise,
- early evidence of benefit or harm of the experimental intervention

2.10 Protocol amendments

Substantial amendments are only implemented after approval of the CEC and CA respectively.

Under emergency circumstances, deviations from the protocol to protect the rights, safety and well-being of human subjects may proceed without prior approval of the sponsor and the CEC/CA. Such deviations shall be documented and reported to the sponsor and the CEC/CA as soon as possible.

All Non-substantial amendments are communicated to the CA as soon as possible if applicable and to the CEC within the Annual Safety Report (ASR).

3. BACKGROUND AND RATIONALE

3.1 Background and Rationale

Head and neck squamous cell carcinoma (HNSCC) is the 6th most common type of cancer worldwide. Of all head and neck cancers, approximately 30% originates from the larynx. In Europe, annually 52.000 patients are diagnosed with cancer of the larynx[8]. About 50–60% of laryngeal squamous cell carcinomas arise from the glottic region[14] and over 80% of these patients present in an early stage[15]. The larynx has important roles in production of voice, coordination of deglutition, and respiration. Therefore, the treatment aim of laryngeal cancer is not only achievement of maximum disease control, but also maintenance of function. Transoral CO_2-Laser Microsurgical Cordectomy (TLM) and radiotherapy are well-established standard treatment modalities for unilateral stage 0-I carcinoma in situ and squamous cell carcinoma of glottic larynx (for convenience, both will be mentioned as SCCGL throughout this protocol)[16,17]. Based on various clinical studies and meta-analyses, functional and oncological outcome (overall survival and local control) after both treatment modalities are comparable[1–5]. However, no properly conducted randomized trials comparing these treatments exist. The only such trial with the endpoint of voice quality had to be prematurely closed due to low accrual[6].

The treatment option varies remarkably in different countries and among institutions. Especially in the case of unilateral SCCGL, other factors such as voice quality, cultural and socio-economic factors, and patients' preference should be considered. Hoarseness is one of the main and early signs of SCCGL, whereas both radiotherapy and TLM influence voice quality by altering the vocal cord motion and anatomy. Another issue worth to consider is the treatment time. Surgery is performed in one day followed by a few days of hospital stay. In contrast, radiotherapy is applied in daily fractions. Although each fraction only takes few minutes, the patients have to visit the radiation oncology department over a course of 4 to 7 weeks, depending on the institutional preference of dose and fractionation. The post-treatment follow-up schedules are identical for both strategies.

The differences between both treatment modalities have been extensively reported. However, either due to no meaningful difference or due to the heterogeneity in the voice analysis techniques, several studies revealed no significant differences[18–20] while better voice quality was reported after radiotherapy as compared with TLM in other studies[6,21–23].

The traditional radiotherapy involves the treatment of the whole larynx over a period of 4 to 7 weeks[24]. Recently, a new radiotherapy technique was introduced by a team of researchers from Netherlands, where the treated target volume consists of involved vocal cord and therefore 8 to 10 fold smaller than the target volumes used for traditional whole larynx irradiation. The treatment is reduced to 16 fractions with higher dose per fraction which corresponds to 3 weeks and a day[7–12]. The results of a prospective cohort (n=30) with single vocal cord irradiation (SVCI) were compared with the results of a historical prospective cohort (n=131) previously treated with whole larynx radiotherapy in the same institute. The median follow-up was 30 months. The voice handicap index (VHI) at all time points beginning from the 6th week after SVCI was significantly superior to the same time points with conventional radiotherapy. Moreover, a comparable local control with SVCI (100%) vs. conventional radiotherapy (92%) was reported at two years, p=0.24[12]. Based on that, the SVCI technique was implemented as a standard approach for SCCGL in many radiation oncology clinics in the recent years, including the centers participating in this clinical trial.

In conclusion, the long-term voice quality and oncologic outcome (survival and local tumor control) are comparable with both TLM and traditional radiotherapy. Furthermore, the new SVCI technique offers a possibly superior long-term voice quality with an at least equal oncologic outcome compared to traditional radiotherapy. With this background, our main aim is to compare SVCI to TLM with the main focus of patient-reported voice quality.

3.2 Investigational Treatments and Indication

With the indication to treat early stage SCCGL in an organ preservation setting, both TLM and SVCI are going to be compared head-to-head, and therefore are considered as investigational treatments.

3.3 Clinical Evidence to Date

In well-recognized international cancer treatment guidelines, TLM and radiotherapy are considered as comparable modalities for the successful treatment of early stage SCCGL[16,17]. Based on various clinical studies and meta-analyses, functional and oncological outcome (overall survival and local control) after both treatment modalities are comparable[1–5]. However, no properly conducted randomized trials comparing these treatments exist. The only such trial with the endpoint of voice quality had to be

prematurely closed due to low accrual[6].

The differences between both treatment modalities have been extensively reported. However, either due to no meaningful difference or due to the heterogeneity in the voice analysis techniques, several studies revealed no significant differences[18–20] while better voice quality was reported after radiotherapy as compared with TLM in other studies[6,21–23]. Nevertheless, most of the literature consists of reports about retrospective case series with the lack of level I evidence.

3.4 Risks / Benefits

Both treatment modalities under investigation are associated with distinct benefits and minimal but potential risks, which are summarized in the table below.

Table 2: Risks and Benefits of Treatments under Investigation

	**Transoral CO_2-Laser Microsurgical Cordectomy**	**Single Vocal Cord Irradiation**
**Certain Benefits**	- Quick (one day treatment followed by few days of hospital stay)<br>- Psychological relief for patients who prefer that their tumor is ‘taken out’<br>- Salvage radiotherapy (in case of tumor recurrence) is relatively easier to perform than vice versa (i.e. salvage surgery after radiotherapy)	- Non-invasive<br>- No requirement for anesthesia<br>- Also applicable for patients with medical conditions hampering operability (irrelevant for the current prospective randomized trial, because medical inoperability is an exclusion criterion)
**Potential Benefits**<br>(primary endpoint under investigation)	- Superior patient-reported voice quality compared to radiotherapy	- Superior patient-reported voice quality compared to surgery
**Possible Risks**	- Need for multiple operations and or adjuvant radiotherapy due to inadequate resection margins<br>Perioperative morbidity<br>- Bleeding<br>- Mild pain<br>- Infection<br>- Prolonged hospital stay<br>Post-operative morbidity<br>- Psychosocial problems associated with the altered voice	Acute radiation toxicity under the treatment, which usually resolves in 2-4 weeks<br>- Increased hoarseness and laryngeal edema<br>- Mild odynophagia<br>- Dermatitis<br>- Fatigue<br>Long-term radiation toxicity (>90 days after treatment)<br>- Functional deterioration of the laryngeal function and aspiration pneumonia, which may eventually require a surgical intervention*<br>- Arteriosclerosis in carotid arteries, which may increase the risk of stroke incidence after 8-10 years*

*: These risks are lower with the SVCI technique compared to the results reported in the literature about whole larynx irradiation. Because, the treatment volumes are 8-10-fold smaller than the conventional whole larynx irradiation, and the normal tissues receive significantly fewer doses (e.g. the carotid arteries receive 5-10-fold less dose)[12].

3.5 Justification of Choice of Study Population

In line with the eligibility criteria, the study population will include patients diagnosed with unilaterally situated stage 0-I SCCGL who are considered as eligible both for radiotherapy and surgery by a multidisciplinary tumor board. Both treatment modalities are well-established standards.

4. STUDY OBJECTIVES

4.1 Overall Objective

The ultimate goal of the study is to compare the treatment outcome of the SVCI and TLM for unilateral stage 0-I SCCGL.

4.2 Primary Objective

Comparison of patient-reported subjective voice quality after TLM and SVCI

4.3 Secondary Objectives

- Qualitative post-therapeutic comparison of the voice between study arms

- Quantitative post-therapeutic comparison of the voice between study arms

- Comparison of the oncological outcome between study arms.

- Assessment of toxicity/morbidity among study arms with the listed toxicity items provided in Appendix A. CTCAE v.5.0 will be used for the classification of toxicities.

4.4 Safety Objectives

- Assessment of toxicity/morbidity among study arms with the listed toxicity items provided in Appendix A. CTCAE v.5.0 will be used for the classification of toxicities.

5. STUDY OUTCOMES

5.1 Primary Outcome

- Average of the VHI assessed at 6, 12, 18, and 24 months

5.2 Secondary Outcomes

- VHI separately assessed at 6, 12, 18, and 24 months
- Perceptual impression of the voice via Roughness – Breathiness – Hoarseness (RBH) assessment at 6, 12, 18, and 24 months
- Quantitative characteristics of voice by means of Jitter and Shimmer (JS), Glottal-to-Noise Excitation Ratio (GNE) and Singing Power Ratio (SPR), which will be assessed at 6, 12, 18, and 24 months
- Loco-regional control of the disease at 2 and 5 years
- Treatment toxicity at 2 and 5 years based on CTCAE v.5.0

5.3 Other Outcomes of Interest

The patient, disease and procedural characteristics will be described.

5.4 Safety Outcomes

Although the safety endpoints within the secondary endpoints will be published only at two time points (2 and 5 years), the toxicity will be systematically assessed during and after the treatment in a more frequent schedule: during the treatment, every 3 months until 24 months, and every 6 months between the 24th and 60th months. Assessment of toxicity/morbidity among study arms with the listed toxicity items provided in Appendix A. CTCAE v.5.0 will be used for the classification of toxicities.

6. STUDY DESIGN

6.1 General study design and justification of design

This is a prospective randomized multi-center open-label comparative phase III study with a superiority design (see Section 11 for the details of statistical considerations). Primary endpoint of this study is patient-reported subjective voice quality between 6 to 24 months after randomization.

The sample size calculation is based on the primary outcome, the VHI at 6 to 24 months (averaged). Based on the literature, we consider the VHI to be comparable between TLM and traditional whole larynx radiotherapy. Therefore, the working hypothesis is that there is a difference between TLM and SVCI in regard to VHI. Al-Mamgani et al. reported standard deviations for VHI ranging from 2 to 10 score points[12]. A difference of 8 points between the two groups are regarded as clinically relevant and a conservative standard deviation of 8 points is assumed. Based on a two-sample means test, 34 patients (17 per group) are needed to detect a difference in VHI at a two-sided alpha-level of 0.05 with a power of 80%.

This sample size calculation is conservative. In the analysis, the average difference over four time points (6, 12, 18, and 24 months) will be modelled and additionally adjusted for the baseline VHI to yield more power. In each center, patients will be enrolled into the study by the local principal investigator. The local PI can be a head and neck surgeon or radiation oncologist. However, before patient accrual, the patient must be informed about the study at least by the attending surgeon and the radiation oncologist. It is highly recommended that the patient is evaluated and informed in a multidisciplinary tumor board setting before the accrual.

Interventions in the trial are considered as non-experimental standard treatments. No diagnostic tool or imaging modality that will be used during the trial is experimental. No interim analysis for futility or safety will be performed.

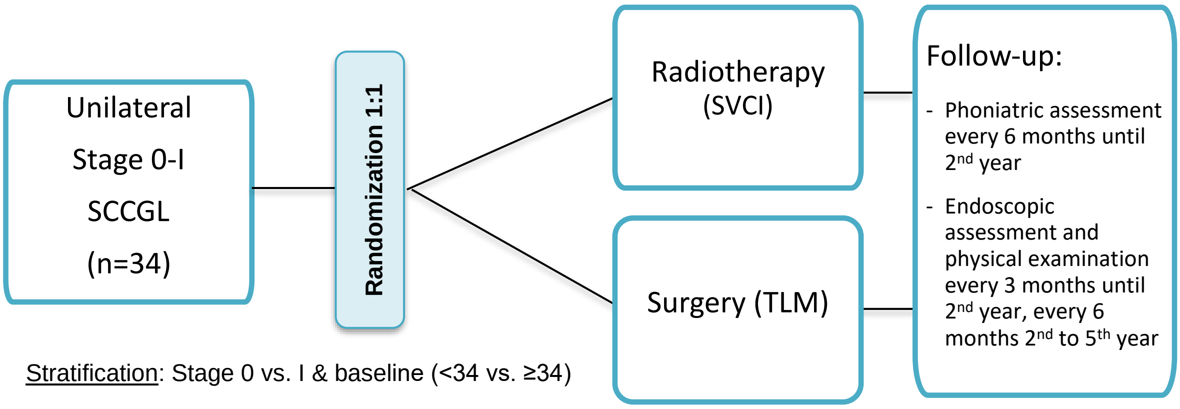

Stratification: Stage 0 vs. I & baseline (<34 vs. ≥34)

SCCGL: Squamous cell carcinoma of glottic larynx (for convenience, both carcinoma in situ and squamous cell carcinoma of glottic larynx are mentioned as SCCGL throughout the protocol); SVCI: Single vocal cord irradiation; TLM: Transoral CO_2-Laser microsurgical cordectomy

6.2 Methods of minimizing bias

6.2.1 Randomization

Please refer to Section 11.2.

6.2.2 Blinding procedures

Due to obvious differences between surgery and radiotherapy, it is not possible to have a blinded design in this study setting.

7. STUDY POPULATION

7.1 Inclusion criteria

1. ECOG performance status 0-1 at the time of registration
2. ≥18 years of age
3. Baseline assessments and documentation of voice quality by means of VHI, JS, RBH, GNE, SPR
4. Histopathologically confirmed, previously untreated unilateral (cT1a or unilateral cTis) stage 0 or I glottic larynx cancer based on the UICC staging system (8th edition)
5. History and physical examination by treating physician (head and neck surgeon and radiation oncologist) within 28 days prior registration
6. The patient must be expected to withstand both study interventions
7. The patient must have undergone panendoscopy with assessment for the feasibility of transoral exposure for resection. Patients without feasible exposure are not eligible
8. Localization of the tumor should allow resection with a minimum of 2 mm macroscopical margin without extension to the contralateral vocal fold, without partial resection of the arytenoid cartilage and without resection of parts of thyroid cartilage (Cordectomy Type I-IV according the classification of the European Laryngological Society)[13]
9. Hemoglobin ≥10 g/dL or 6.2 mmol/L (Note: The use of transfusion to achieve Hgb ≥10 g/dL is acceptable) within the 28 days prior to accrual
10. Women with child-bearing potential and using effective contraception, and not pregnant and agree not to become pregnant (see section 8.6) during participation in the trial and 30 days after radiotherapy. A negative pregnancy test before inclusion (within 28 days) into the trial is required for all women with child-bearing potential. Men agree not to father a child during participation in the trial and 30 days after radiotherapy.
11. Written informed consent, signed by the patient and the investigator.

7.2 Exclusion criteria

1. Infection hampering the voice quality at the time of voice assessment
2. Involvement of the anterior commissure by the tumor
3. Previous oncologic surgery with curative intent (exception: excisional biopsies resulting in unacceptable close R0 or R1/R2 margins may be included) or radiotherapy to larynx
4. Synchronous or previous malignancies. Exceptions are adequately treated basal cell carcinoma or SCC of the skin, or in situ carcinoma of the cervix uteri, low-risk prostate cancer or breast with a cancer-free follow-up time of at least 3 years, or other previous malignancy with a progression-free interval of at least 5 years
5. Co-existing disease prejudicing survival (expected survival less than 6 months)
6. Active bacterial or fungal infection requiring intravenous antibiotics at the time of registration
7. History of any voice disorders (not related to the SCCGL) lasting longer than 3 weeks
8. Illness requiring hospitalization or precluding study therapy within 28 days before registration
9. Presence of any psychological, familial, sociological or geographical condition potentially hampering compliance with the study protocol and follow-up schedule; those conditions should be discussed with the patient before registration in the trial

7.3 Recruitment and screening

Patient registration/randomization will only be accepted from authorized investigators.

Prior to registration, the following steps have to be taken:

- Fill in the patient screening (used for monitoring potentially eligible patients, and will be destroyed after the end of the accrual period. Screening list is not a part of the CRFs), enrollment and identification lists.
- Check the eligibility criteria
- Obtain signed and dated written informed consent from the patient prior to any protocol-specific procedure according to ICH/GCP and local guidelines.
- Patients must complete the phoniatric assessments per protocol

Only electronic case report forms (eCRF) will be used. The use of worksheets is allowed if the copies

of the templates are documented in the trial master file (TMF). The used worksheets must be kept with the patient charts.

Registration is done via Internet ‘https://secutrial.insel.ch’. In case of problems investigators can phone the study coordinator from Monday through Friday. For technical difficulties, investigators are recommended to contact data management of CTU Bern

Phone: +41 31 631 33 72

Fax: +41 31 631 33 73

E-mail: datamanagement@ctu.unibe.ch

In order to receive authorization for online registration/data entry, sites must send a copy of the completed staff list to the CTU Bern. Login details for the online database will be sent to authorized persons.

7.4 Assignment to study groups and randomization

For each patient who has signed the informed consent form, dedicated study personnel at each study site will access the electronic case report form (eCRF) at https://secutrial.insel.ch and generate a new patient record.

Each patient is identified by an alphanumeric unique patient code which is a combination of the applicable center number and a subject number. The center number is assigned by the sponsor-investigator to the investigative site. A list with patient numbers assigned to patient identifying information will be kept in the investigator site file at each site, with access restricted to study personnel only.

After eligibility criteria have been reviewed at baseline, patients will be randomized to one of the following two treatment arms:

Arm A: Transoral CO_2-Laser Microsurgical Cordectomy

Arm B: Single Vocal Cord Irradiation

7.5 Criteria for withdrawal / discontinuation of participants

Patients have the right to refuse further treatment for any reason and at any time. Patients who decide to withdraw from the trial should be asked whether they also want to withdraw their consent for their data to be used for the follow-up assessments. If allowed by the patient, the data will be used for further analysis and anonymized thereafter. For the patient’s security, a last examination should be performed.

Patients may be withdrawn at any time from trial treatment at the discretion of the investigator due to a serious adverse event or based on any other relevant medical condition. The patient will then be transferred to the follow-up phase.

8. STUDY INTERVENTIONS

8.1 Identity of Study Interventions

Despite being considered as standard treatment modalities, both study interventions (TLM and SVCI) in this study are considered as study interventions and subject to equally-weighted efficacy and safety assessments.

Both study interventions must be started within 4 weeks and ideally within 2 weeks from the date of patient registration.

The diagram hereunder must be used by the physician conducting the panendoscopy to document the lesion. This diagram must be submitted to the:

1. Surgical and pathology teams during the surgery and postoperative period (surgery arm – Arm A);
2. Radiation oncology team for the SVCI planning (radiation arm – Arm B)

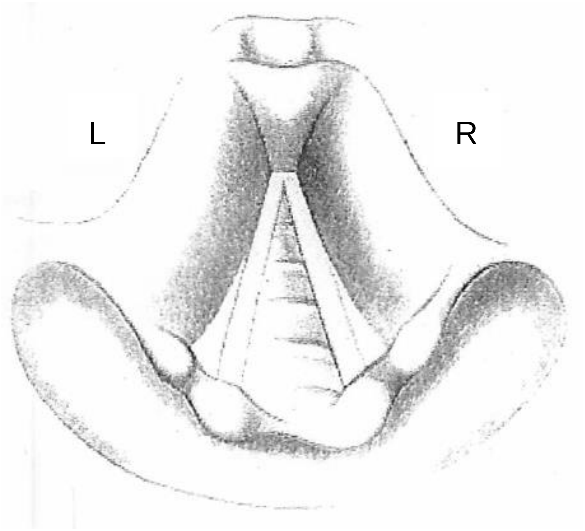

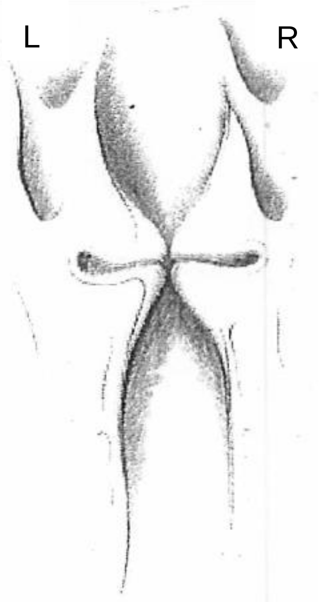

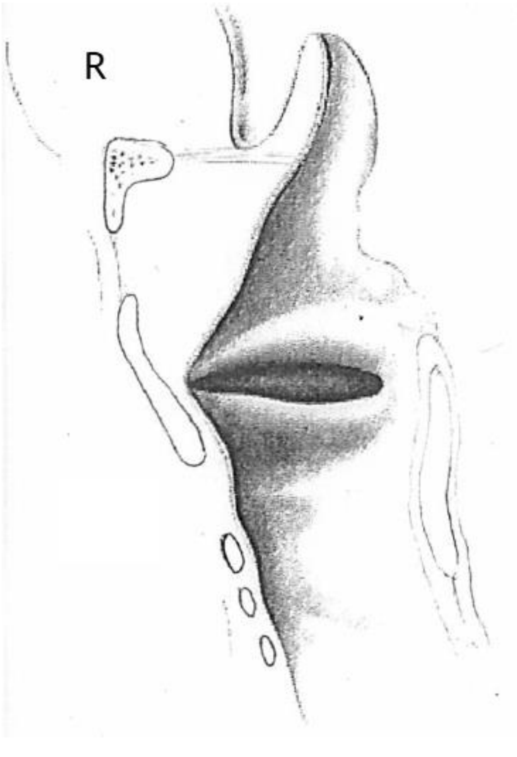

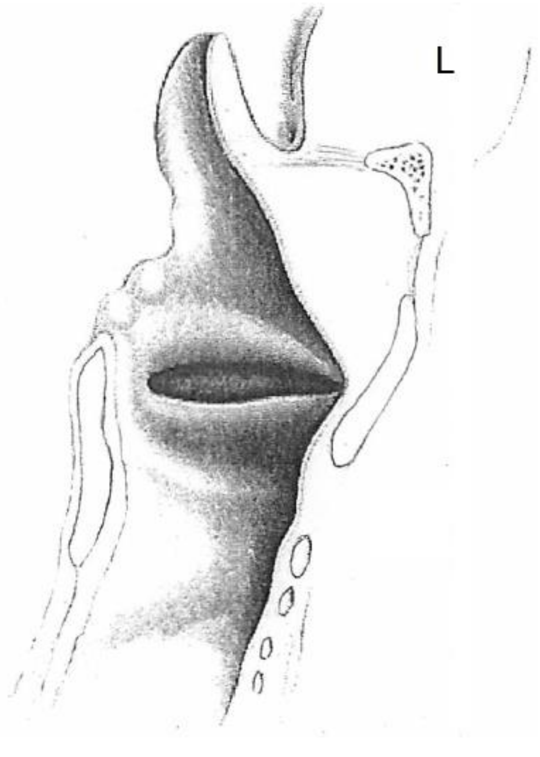

8.2 Transoral CO_2-Laser Microsurgical Cordectomy (TLM) in Arm A

8.2.1 Preoperative Assessment

To determine the feasibility of the surgery under general anesthesia, the anesthesia team will perform a preoperative assessment as necessary. In case of refusal due to any medical conditions, the patient has to be withdrawn from the study and treated off-protocol. A panendoscopy with assessment of trans-oral exposure for resection has to be performed before accrual. In case, the glottic space cannot be correctly exposed for TLM, the patient cannot be included in the study.

8.2.2 Definition of Technique and Resection Margins

The TLM has to be performed using a CO_2 laser, coupled to an operative microscope, at 2-8 W in ultrapulse mode. The type of cordectomy performed must be mentioned using the following classification according the classification of the European Laryngological Society[13]:

Type I: subepithelial resection respecting the vocal ligament
Type II: subligamental resection
Type III: partial resection of the vocalis muscle
Type IV: total resection of the vocal fold

The type of resection chosen should provide complete removal of the primary lesion with negative margins.
Surgery will generally be performed within 3 weeks after randomization and not more than 6 weeks after panendoscopy.

The extent of the cordectomy must include a complete anterior, posterior, inferior and supero-lateral mucosal and deep soft tissue margin.

<u>Tis:</u>

The surgeon should aim to achieve a resection of tumor and surrounding structures, with an attempt made to achieve at least a 1 mm macroscopical mucosal margin, with a minimum of 0.5-mm microscopic margins. These recommendations are subject to quality assurance (QA) review. The specimen should be oriented by the surgeon as per local practice.

<u>T1a:</u>

The surgeon should aim to achieve a resection of tumor and surrounding structures, with an attempt made to achieve at least a 2-mm macroscopical mucosal margin, with a minimum of 1-mm microscopic margins. These recommendations are subject to QA review. The specimen should be oriented by the surgeon as per local practice.

8.2.2.1 <u>Definition of Margins and Postsurgical Management of Close or Positive Margins</u>

Positive margins are defined by in situ or invasive carcinoma in contact with the margins. Close margins are defined by in situ or invasive carcinoma <0.5 mm and <1 mm, respectively. Transoral re-resections are required in case of R1 or close-margin to convert the patient to an R0-status.

• R0/clear margin is defined as $\geq$0.5 mm (Tis) and $\geq$1 mm (T1a) in the final specimen

• Close margin is defined as >0 mm to <0.5 mm (Tis) and >0mm to <1 mm (T1a) in the final specimen.

• R1/positive margin is defined as 0 mm in the final specimen.

In case of either close or positive margins it is mandatory to perform a second intervention **within 4 weeks after primary surgery with an attempt to clear the margin**. This will consist of a re-resection in the area of the involved margin. This is subject to QA. If a surgery is not done to clear the margin in case of a positive or close margin, further treatment with radiation therapy will be proposed to the patient (see the next section below).

Standard terminology for the margins of the specimen will include the following: anterior, posterior, inferior, superior, lateral. The final margins are determined from the primary resection specimen. It is therefore of great importance to adequately arrange the pieces with the correct orientation prior to sending to the pathologist.

8.2.3 Post-surgical Management of Close or Positive Margins

Re-resections are strongly recommended in order to achieve adequate resection margins and to avoid a multimodal treatment.

Close/positive margins: Close/positive margins are defined to be between 0 mm to <0.5 mm (Tis) and 0 mm to <1 mm (T1a) of distance between the tumor front and the surgical cut in the primary specimen. In this case a re-resection with an attempt to clear this margin should be performed, unless the patient refuses or has other adverse features requiring postoperative radiation therapy only. If this second margin shows no evidence of disease, the final margin is considered to be clear.

In case of inadequate resection margins despite of surgical efforts or if the patient refuses further surgery, an **adjuvant radiotherapy** shall be considered at the discretion of the institutional tumorboard and started within 5-6 weeks after surgery or re-resection, respectively. The technical details of the adjuvant radiotherapy are out of the scope of this study. The treatment planning and the conduction of the adjuvant radiotherapy must be carried out according to the institutional local standards. However, it is not allowed to use a fraction dose lower than 1.8 Gy or higher than 2.25 Gy, and a total dose lower than 60 Gy.

8.2.4 Post-operative Care

In general, the standard of care per institution will be followed in terms of monitoring for:

- Airway management
- Bleeding after surgery
- Oral feeding and swallowing function

In more detail, the post-operative care aims at most rapid recovery of oral feeding. Mobilization should take place at the day of surgery, if permitted. The duration of hospitalization is at the discretion of the surgeon and should be for the period of risk for post-operative complications, i.e., post-operative bleeding. Postoperative antibiotic therapy is not amended.

8.2.5 Expected Complications / Surgical Adverse Events of TLM

In this study, surgical complications will be noticed within 30 days after primary and also re-resection TLM. Day 0 is the date of surgery.

Complications at the primary site after TLM are generally rare, but the most common ones consist of:

- Post-operative bleeding
- Airway compromise

Other known and common complications of any surgery include:

- Complications related to general anesthesia
- Pain
- Wound site infection
- Thromboembolic events
- Pneumonia
- Single or multi-organ failure

Surgical Adverse Events will be recorded using the Clavien-Dindo classification of surgical complications[25] (Appendix B). Serious adverse events (SAE) will be documented reported via SAE procedure (Section 10).

8.2.6 Standardization of the Surgical Technique

8.2.6.1 Documentation of the Surgical Technique

Operative reports and pathology reports of each patient must be available for review. Margin status, in case of positive or close margins re-resection protocols, pathology reports of follow-up interventions and surgical complications are subject to QA (see Section 8.2.2). The reference diagram on Section 8.1 must be used by the surgeons for documentation of the lesions assessed during endoscopy and intraoperatively, and submitted to the pathologist together with the operation report.

8.2.6.2 Documentation of Margins

Margins are determined in the primary resection specimen. Additional frozen section cannot be added later to calculate the margin. However, the primary tumor resection can be performed in multiple pieces.

If this is the case, it should be mentioned in the operation report and the pieces have to be oriented with ink or sutures. The pathologist needs to photograph the specimen in order to show the blocks selected for cutting.

8.2.6.3 *Pathology Quality Assurance (QA): Specimen Handling and Labeling*

The overall approach to specimen handling and reporting is based on that outlined in the Dataset for Histopathology Reporting of Laryngeal Cancer, College of American Pathologist, 2017: https://www.cap.org/protocols-and-guidelines/cancer-reporting-tools/cancer-protocol-templates

All specimens must be oriented by the surgeon as per local practice, preferably pinned to a flat surface with a diagram of the vocal fold for orientation. The specimen can be labeled either with sutures or with ink. Every specimen should be sent with a figure indicating the orientation of the specimen using common terms such as “anterior, posterior, inferior, superior-lateral mucosal and deep soft tissue margin”.

The handling of TLM specimens requires close collaboration between the surgeon and pathologist. The resection should be orientated by the surgeon, as per local practice, but this must allow for identification of all margins. The specimen must be formalin-fixed within 2 hours and subsequently paraffin-embedded within 48 hours according to standard procedure.

8.2.6.4 *Photography of Resected Specimen*

A photograph of every specimen needs to be taken by the pathologist prior to cutting to demonstrate the final resected specimen properly oriented and labelled, and demonstrating adequate margins. This should be done as well to allow for a record in any case. If the main specimen is presented in multiple parts, these must be presented in a manner which allows orientation to each other and identification of the margins.

8.2.6.5 *Core Data to be Reported in the Local Pathology Report*

- Operative procedure
- Specimen submitted
- Tumor localization and laterality
- Tumor dimension
    - Macroscopic diameter in millimeters should be used unless the histological extent measured on the glass slide is greater than what is macroscopically apparent, in which case the microscopic dimension is used.
    - In case multiple pieces are received, the aggregated dimension can be used.
    - The preoperative imaging and/or endoscopy measurement and surgical findings can be used to indicate a more precise tumor dimension.
- Histological tumor type based on WHO classification
- Histological grade
- Perineural invasion
- Lymphovascular invasion
- Margin status (see the section Documentation of Margins in the previous page)
- Ancillary studies, if performed
- Pathological staging

8.2.7 Quality Assurance (QA) Procedures (Adequacy of the Oncologic Surgery)

For each participating center, a central QA review will be performed after receiving the complete surgery and pathology related patient data, formalin-fixed paraffin-embedded tissue* and the corresponding photograph (see the relevant section above) of the first patient treated in Arm A and afterwards in sets of 3 patients for the whole trial. The final cases will be reviewed within 3 months after this study has reached the target accrual or as soon as complete data for all cases enrolled has been received, whichever occurs first.

No further patient enrollment to the study in a participating center will be allowed, until the central QA of first case in Arm A is successfully completed.

Only one further patient may be enrolled to the study in a participating center, until the central QA of a batch of last set of 3 patients in Arm A is successfully completed.

*: Instead of sending paraffin-embedded tissue, digital pathology solutions may be used upon agreement with the central pathology review team.

8.2.7.1 *Goals of Surgical QA Procedures*

- To assure safety of patients undergoing surgery
- To assure the quality of oncologic surgical principles

8.2.7.2 *Surgical and Pathological Protocol Compliance Criteria*

The central review process will be done to assess compliance and accuracy of data. This will be based on the case report forms and data from operative and pathology reports. A separate technical document for the quality control procedure will be available. A surgical and pathology team will then determine:

- Acceptable minor variations
- Acceptable major deviations
- Unacceptable deviations/protocol violations

Quality indicators will include:

- Margin status (clear vs. positive/close) invasion
- Minimal distance from tumor to surgical margin, both mucosal and deep.
- Type of peri- and postoperative complications
- Frequency of revision procedures

Acceptable (minor) Deviations

- Suboptimal resection with close margin

Unacceptable (major) Deviations

Those deviations that affect patients' safety/outcome, which may result in an institution being suspended from further participation in the study, such as:

- Positive margin without re-resection within 4 weeks or adjuvant radiotherapy within 5-6 weeks after the last surgical intervention
- Bleeding requiring operative control exceeding 20%

Not reporting cases of major complications like postoperative airway obstruction, bleeding and revision surgery

8.3 Single Vocal Cord Irradiation (SVCI) in Arm B

8.3.1 Equipment and Tools

The participating centers must have defined and implemented a RT-QA program for the commissioning and operation of the linacs, complying with current national or international recommendations. The participation in regular external dosimetry audits (EDA) to assess beam output is mandatory.

The participating centers must have defined and implemented a RT-QA program for patient specific RT treatment plan verification. The centers must provide evidence for a successful implementation and usage of RT treatment planning, application and verification.

Proofs for the implementation of the RT-QA programs and the successful participation in the external audits must be supplied and a dummy run is required for this trial.

8.3.2 General Considerations for Treatment Planning

Inverse planning techniques (as defined in ICRU 83 report) are required, using a dose calculation algorithm that takes into account tissue heterogeneity. The target volume will be defined by both imaging and clinical evaluation.

8.3.3 Patient Positioning

The patient should be simulated and treated in supine position with arms on the side of the trunk, adequately positioned and fixated according to the routines at each treatment center. In order to minimize the swallowing motion, dorsal hyper-reclination of the head (within the range of patient's comfort) is recommended. The positioning should be same during the planning and treatment. The use of thermoplastic masks covering at least the head and neck area is mandatory. Masks additionally covering the shoulders are strongly recommended. Orthogonal laser beams should be used during the acquisition of planning CT and treatment set-ups. Positions of laser beams on the thermoplastic masks and patient's sternum and shoulders should be marked accordingly by securing their durability until the end of the treatment.

8.3.4 Simulation (Patient Image Acquisition)

A volumetric treatment planning CT scan is required to define the target volumes; using a set of slices extending at least from the level or the base of skull to the suprasternal notch. In case of no contraindication, it should be performed with i.v. contrast so that the major vessels of the neck are easily visualized. Contrast corrections are neither necessary nor recommended. The treatment planning CT scan must be performed with the immobilization device in place and in the treatment position.

A respiratory-gated 4D CT scan with 1 mm slice thickness and an in-plane resolution of at least 1x1 mm^2 will be acquired. From the 4D-CT scan a 3D maximum intensity projection (MaxIP) and a 3D mean intensity projection (MeanIP) will be generated.

8.3.5 Definition of Volumes

For a practical quality control processes and volumetric/dosimetric analyses, the given nomenclature for the target volume and OAR will be used in all study centers.

8.3.5.1 Target Volumes

The different volumes of interest should be defined in agreement with ICRU Reports 50, 62 and 83, namely:

- Gross Tumor Volume (GTV)
- Clinical Target Volume (CTV)
- Planning Target Volume (PTV)

GTV and CTV will be delineated on the MeanIP, as mentioned in the previous Section 8.3.4. CTV = GTV with a concentrically isotropic margin of 3 mm in all directions, or the entire ipsilateral vocal cord in case the GTV cannot be clearly delineated. To overcome this problem, it is strongly recommended to depict the tumor extension together with the head and neck surgeon on photographs taken during the endoscopy (if possible endoscopy under general anesthesia). The use of other imaging modalities (e.g. magnetic resonance imaging) to define the GTV is left to the discretion of the responsible radiation oncologist.

In the axial plane after the extension of the margins, CTV can include the paraglottic space, the anterior commissure for anterior vocal cord tumor, the anterior part of the contralateral vocal cord for tumor extending towards the anterior commissure (within 2 mm), and the arytenoid cartilage (at least its vocal process) for tumor extending to the posterior vocal cord (within 2 mm or involving the ipsilateral arytenoid) but excludes the thyroid cartilage and the air cavity (visualized in CT lung windowing as previously described[26]).

In the cranio-caudal plane, the CTV includes the subglottic and the supraglottic aspects of the vocal cord. In case the target volume(s) in the MaxIP was not fully included in the initial contours, these contours will be extended to ensure enclosure. This was reported to occur rarely and if present, deviations are usually below 1 mm[12].

The following anisotropic PTV margins will be used around the CTV

- Left - right: 3 mm
- Cranial - caudal: 5 mm
- Anterior - posterior: 3 mm

8.3.5.2 Organs at Risk

All organs at risk (OAR) except for swallowing muscles (Cricopharyngeous muscle, Middle constrictor muscle and Inferior constrictor muscle) listed under Section 8.3.6.1 will be delineated on the MeanIP according to the international consensus guidelines by Brouwer et al. 2015[27]. The swallowing muscles will be delineated as defined by Christianen et al. 2011[28]. The only exception is the OAR "Larynx", which shall include the epiglottis, the whole soft tissue and cartilaginous structures, but excluding the hyoid bone.

8.3.6 Treatment Technique, Treatment Planning and Dose Prescription

Radiotherapy should be delivered with external megavoltage energy photon beam therapy with the inverse planning technique. The position of the patient should be verified with volumetric imaging (VIM) according to equipment in each treatment center (e.g. cone-beam CT, fan-beam CT or on-board magnetic resonance imaging). The use of online patient positioning and re-positioning (if necessary) is mandatory.

Treatment planning shall be performed using a state-of-the art treatment planning system (TPS). The TPS must permit an optimization of dose-volume parameters for the PTV and OAR. The dose calculation algorithm shall be model based and take into account secondary electron and photon transport. It must include a heterogeneity correction. Algorithms based only on path length scaling are not permitted. The participating centers must have implemented a RT-QA program for TPS commissioning and use, complying with current national or international recommendations. The CT patient datasets must cover the whole irradiated area plus a margin extending at least to the base of skull and suprasternal notch in cranio-caudal direction. The dose must be calculated on the whole MeanIP CT dataset. The documentation of the patients' treatment plans must include:

- Dose distributions in at least three representative orthogonal planes
- Dose Volume Histogram (DVH) of target volumes and OARs
- List of parameters for all treatment and setup fields
- Prescription dose and normalization method

The following planning aim will be pursued: full coverage of the PTV with at least 95% of the prescribed dose and a maximum (0.03 cc) PTV dose of <107%: 16 x 3.63 = 58.08 Gy in 5 fractions per week (equivalent dose $EQD2_{\alpha/\beta=10Gy}$ = 66 Gy, $EQD2_{\alpha/\beta=3Gy}$ = 77 Gy) using 5 to 9 static IMRT beams as described before[7,11,12]. Alternatively, VMAT plans fulfilling the same criteria may be applied. Refer to Section 8.3.7 for special considerations during the treatment delivery of static IMRT and VMAT plans. Additionally;

- 6-MV photons will be used.
- No bolus will be used.
- For the IMRT plans, the use of non-co-planar beam angles will be allowed as described by Osman et al. (2012)[7].
- Dose calculation will be performed with dose calculation algorithms that account for heterogeneities and consider lateral scatter and electron transport, e.g.:
    - Convolution/Superposition algorithms like CC (collapsed cone) of Pinnacle or Oncentra Masterplan,
    - AAA (anisotropic analytic algorithm) of Eclipse,

- Superposition of CMS/XiO,
- Monte Carlo,
- Acuros XB (Boltzmann Solver) of Eclipse,
- or equivalent.

The sections below specify the treatment planning objectives and constraints.

8.3.6.1 Prioritization for Planning

1. Spinal cord (SpinalCord)
2. PRV Spinal cord (PRV_SpinalCord, defined as SpinalCord + 5 mm isotropic margin)
3. PTV
4. Brachial plexus (BrachialPlexus)
5. Contralateral vocal cord (CL_VocalCord)
6. Contralateral arytenoid (CL_arytenoid)
7. Ipsilateral arytenoid (IL_arytenoid)
8. [No specific ranking]
    - Cricopharyngeous muscle (Cricopharyng)
    - Middle constrictor muscle (Mid_Constrictor)
    - Inferior constrictor muscle (Inf_Constrictor)
    - Larynx (together with the cartilaginous structure)
    - Supraglottic larynx (Supra_Larynx)
    - Ipsilateral carotid artery (IL_carotid)
    - Contralateral carotid artery (CL_carotid)
    - Thyroid gland (Thyroid)

8.3.6.2 Dose-volume objectives/constraints for target volumes and OARs

Table 3: Dose-volume objectives

Parameter	Per Protocol	Variation Acceptable	Deviation Unacceptable
$D_{99\%}$ of PTV	58.08 Gy	≥55.176 Gy (95%)	<55.176 Gy (95%)
$D_{1\%}$ of PTV	≤60.98 Gy (105%)	≤62.15 Gy (107%)	>62.15 Gy (107%)
$D_{0.03\,cc}$ of PTV	≤62.15 Gy (107%)	≤63.89 Gy (110%)	>63.89 Gy (110%)
$D_{0.03\,cc}$ outside the PTV (Body-PTV)	≤60.98 Gy 105%	≤62.15 Gy (107%)	>62.15 Gy (107%)

Table 4: OAR Constraints

Volume	Constraint[¥]
SpinalCord*	D_{max} ≤30 Gy
PRV_SpinalCord*	D_{max} ≤35 Gy
CL_VocalCord	D_{mean} ≤50 Gy
Larynx	D_{mean} ≤40 Gy
CL_arytenoid	D_{mean} ≤40 Gy
IL_arytenoid (if not overlapping with PTV)	D_{mean} ≤52 Gy
Mid_Constrictor	D_{mean} ≤40 Gy<br>V43 Gy ≤33%<br>V50 Gy ≤15%
Inf_Constrictor	D_{mean} ≤40 Gy<br>V43 Gy ≤33%<br>V50 Gy ≤15%
Supra_Larynx	D_{mean} ≤20 Gy
Cricopharyng	D_{mean} ≤40 Gy<br>V43 Gy ≤33%<br>V50 Gy ≤15%

BrachialPlexus*	D_{max} <45 Gy
IL_carotid	D_{max} <30
CL_carotid	D_{max} <15 Gy
Thyroid	D_{mean} <15 Gy

CL: contralateral; IL: ipsilateral; PRV: planned risk volume

*: constraints mandatory to meet; ¥: Doses to the OARs should be further reduced by the treatment planner than the values given here, if feasible and if the PTV coverage is not compromised.

8.3.7 Treatment Delivery and Verification with Online Imaging

Patients will be instructed not to swallow during VIM acquisition and beam delivery. Therefore, they have to be informed about the start and the end of each beam-on time. If they feel the urge to swallow, they will instruct the RT therapists (e.g. with a predefined hand gesture) to interrupt and repeat the image acquisition / pause the irradiation. The patients will be reminded about this before each treatment fraction. Alternatively, a hand-held button to signal the RT therapists may be handed over to the patients, which is left to the discretion of the local investigators. The swallowing action is expected to last between 1 to 6 seconds[29], which concerns the pauses in the image acquisition and irradiation. In case of swallowing without the option to pause the scan, the patients shall inform the RT therapists and the scan shall be repeated. The number of interruptions and repeated scans will be documented.

For example, in terms of swallowing action, the static-field IMRT has the advantage of allowing the patients to swallow between the gantry angles. On the other hand, the VMAT has the advantage of shortening the overall fraction time. However, the duration not to swallow is longer. Therefore, patients' instruction and bilateral communication between the RT therapists and the patients is a key element for the success of the treatment.

The slice thickness and the resolution of volumetric on-board imaging shall be selected to best-match the parameters of the planning CT. **<u>As an example</u>**, below are the parameters set by the investigators from Erasmus MC for their equipment (Elekta Oncology Systems, Crawley, UK), where the SVCI technique was developed:

- *1 mm slice thickness (at least 1mm² in-plane resolution)*
- *100 kV, 36.1 mAs*
- *-100º to +100º gantry angles*
- *The scan time is expected to average out the small breathing motion of the vocal cords in the VIM (cone-beam CT)*
- *In case of swallowing, the patients shall inform the RT therapists, and the scan shall be repeated. The number of interruptions and repeated scans will be documented.*

8.3.7.1 <u>Positioning and VIM matching</u>

- This section applies to cone- or fan-beam CT equipment. Centers using MRI-mounted irradiation equipment shall contact the PI in order to define a device-specific protocol.
- VIMsetup: Volume of interest (VOI) encompassing the thyroid cartilage, while excluding the hyoid bone and vertebra will be used for automatic registration. A pre-contoured volume of interest (VOI_MATCH) encompassing the cartilaginous structures of the larynx without a margin will be used for automatic matching. It is allowed to use a specific range of Hounsfield units to further optimize the automatic matching algorithm at the discretion of the treatment team. Each matching will be visually verified and manually corrected, if necessary.
- Couch correction will be done online, only when the translational and rotational motion in one or more directions is at least 1 mm and/or 1 degree. The use of a robotic treatment couch with 6 degrees of freedom is allowed and recommended. However, <u>the roll movement of the 6D-couch may be disabled</u>, if the treating team has unfavorable experience with over-rolling due to gravitational pull or voluntary/involuntary contra-"correction" action towards the opposite direction by the patient triggered by her/his discomfort.
- VIMpre: For each patient, an additional VIM will be performed after the VIMsetup +/- couch correction in the first 4 fractions. Only if VIMpre shows a motion of ≥2 mm and or 2 degrees in any direction, a second couch correction will be applied. The acquisition of VIMpre's will continue until a second couch correction is no more required in the last subsequent 3 fractions for the treated patient.
- VIMpost: At the end of each fraction, another VIM will be acquired to verify that intrafraction

target motion was within the applied CTV - PTV margin. If deemed necessary, a new plan with adapted PTV margins may be required.

In the study published by Kwa et al. (2015)[11], in 588 of 672 fractions (87.5%), couch corrections were performed based on VIMsetup. Displacements in VIMpre gave rise to a second couch correction in 19 fractions (2.8% of the fractions). In 27 fractions (4.3%), target displacements derived from the VIMpost scans exceeded the applied CTV-PTV margins: 4, 16, and 7 times in the LR, CC, and AP directions, respectively. The largest observed deviation was 7 mm in the caudal direction. For 30 patients (71.4%), CTV-PTV margins were never exceeded. For the other 12 patients, the margins were exceeded at least once: 1, 2, 3, 4, and 5 times for 5, 3, 1, 2, and 1 patient or patients, respectively. The imaging dose from each cone-beam CT scan was estimated to range between 0.2 cGy to 7 cGy per scan[30].

8.3.8 Fractionation Schedule

The dose will be delivered in consequent 16 single day fractions on 5 days per week except for the weekends. Prolongation of overall treatment time should be avoided. In cases of unplanned treatment breaks or of a holiday, it is not allowed to keep the overall treatment time by giving the patient two fractions per day.

Treatment breaks should ideally not exceed five treatment days at a time and ten treatment days total. Treatment breaks should be allowed only for resolution of severe acute toxicity at physician's decision and/or for intercurrent illness and not for social or logistical reasons. Any treatment break(s) exceeding two treatment days for other than medical/technical reasons, official holidays or patient's decision will be considered as minor protocol deviation.

8.3.9 Facility Questionnaire and Dosimetry Audit

All participating institutions must perform a Facility Questionnaire (FQ) that will collect information about the participating institution and about the methods to be applied for this specific study. The FQ will be reviewed by the RT-QA group.

These institutions must provide evidence for a successful implementation and usage of the RT technique which corresponds / is relevant to the treatment described by the protocol. Further, the implemented quality assurance strategies to ensure correct delivery of the treatment described by the protocol must be specified. In particular, QA procedures of the imaging system (e.g. CBCT) aiming to a coincidence between imaging and machine isocenter have to be provided. Details will be enquired in the FQ.

All participating centers must provide a valid External Dosimetry Audit (EDA) for the machine(s) planned to treat the study patients, which shall be not older than two years. An updated EDA shall be submitted once every two years.

8.3.10 Radiotherapy Quality Assurance (RT-QA)

The purpose of the RT-QA protocol is to ensure uniformity of all radiotherapy data for all patients.

The working group for radiotherapy quality assurance (RT-QA group) consists of all panel members for radiotherapy and radiotherapy quality assurance. Radiotherapy related treatment information and other relevant documentation for each patient shall be sent to the RT-QA group at least 5 working days before treatment start. Positive or negative feedback shall be provided by the RT-QA group at least 2 working days before treatment start. Considering the prognosis of early stage SCCGL this study-specific delay is not expected to have a substantial impact on the treatment outcome.

After the treatment of first 2 patients in Arm B (radiotherapy) in each center, an on-site or online monitoring by the RT-QA team will be carried out to perform an offline review of the IGRT of these 2 patients' treatment courses. No further patients in this center will be accrued before the successful completion of the on-site monitoring.

8.3.11 Dummy Runs

A two-step dummy run will be performed before the start of the trial in each participating institution. CT images of a HNSCC patient will be made available to participating centers from the RT-QA group. Delineation of target volumes and OARs according to the study protocol will be performed at each center, as well as a treatment plan according to the study protocol. The first step of the dummy run involves the generation of the volumes and uploading the data to the central database. The contours will be reviewed by the physicians and the physicists of the RT-QA group. In case of major deviations, these will be

communicated back to the institution, and a revised version shall be submitted until no major deviations persist. Followed by the approval of the volumes, the second step involves the generation of the RT plan. It shall use the approved structures and the treatment technique per protocol. The treatment plan shall fulfill the requirements defined in the previous sections of the protocol. A patient-specific pretreatment QA verification for the generated RT plan shall be performed and documented appropriately. In case of major deviations, these will be communicated back to the institution, and a revised version of the treatment plan shall be submitted until no major deviations persist.

In the following circumstances, the RT-QA group may ask the local center to repeat the dummy run process:

- Permanent change of the authorized radiation oncologist (temporary absences due to holidays, health reasons etc. do not count)
- An interval of 1 year without a new study patient treated

8.4 Compliance with study intervention

The compliance with the study interventions will be assured via regular data monitoring and on-site monitoring, which will be coordinated by the CTU Bern, and via individual patient checks and data-flow as mentioned in the previous sections above.

8.5 Data Collection and Follow-up for withdrawn participants

Participants withdrawn from the trial before any treatment will undergo surgery or radiotherapy as planned by the tumor board. Patients may change their mind and prefer a treatment other than the study arm they were randomized to, or no treatment at all.

If a patient is withdrawn during or after the commencement of any study treatment, he/she will be asked, whether their study-specific data collected so far may be used. The data will be anonymized after the analysis.

A patient may also be withdrawn from the study by the local principal investigator, if the patient's participation in the study deemed to bear any additional risk for the well-being of the participant.

8.6 Trial specific preventive measures

None except for the preventive measures against pregnancy as mentioned under the eligibility criteria and the pregnancy test before the recruitment (and until the end of 3 months after treatment in case of suspicion for pregnancy). During the treatment and within the first 30 days of the follow-up phase, patients have to take contraceptive measures against pregnancy. Effective contraceptives can be hormonal (e.g. contraceptive pill, injections, or implants) or mechanical (e.g. condom, intrauterine pessary, diaphragm, cervical cap or another device). When using mechanical contraceptives, two methods of mechanical contraception have to be used. When using hormonal contraceptives, one additional method of mechanical contraception has to be used.

8.7 Concomitant Interventions (treatments)

No study-specific concomitant interventions are planned. However, symptom-oriented medications are allowed (e.g. pain medications to alleviate post-operative pain, steroids to alleviate laryngeal edema during radiotherapy).

9. STUDY ASSESSMENTS

9.1 Study flow charts

Table 1: Summary flowchart

Study Periods ➔	Screening and Enrollment	Treatment Period	Follow-up until 60 months <u>after enrollment</u>
Time unit ➔	Days	Weeks	Months
Patient Information and Informed Consent	-28 – 0		
Medical history and demographics	-28 – 0		
Panendoscopy, assessment of exposure and staging according to institutional local standards	-28 – 0		
Eligibility Criteria Check	-28 – 0		
SCCGL-oriented physical examination	-28 – 0		
VHI[#], RBH, GNE, SPR, JS	-28 – 0		(1.5)[#], 6, 12, 18, 24 (+/-15 days)
Enrollment and randomization*	0		
Endoscopic assessment of the index tumor, symptom oriented physical examination	-28 – 0		every 3 months (+/-15 days) until 24th month<br>every 6 months (+/-15 days) between 24th and 60th months
Evaluation of actual smoking status and toxicity according to CTCAE v.5.0 (Appendix A)	-28 – 0 (baseline smoking status and toxicity[¥])	weekly during radiotherapy and within one week of surgery: no need to document except for SAEs	
Pregnancy test for women with child-bearing potential	-28 – 0	3 – 7[Ψ]	until 3rd month[Ψ]
Arm A: Transoral CO_2-Laser Microsurgical Cordectomy[∇]		day of surgery +/- day of re-resection	
Arm B: Single Vocal Cord Irradiation[∇]		3 (16 fractions)	

GNE: Glottal-to-Noise Excitation Ratio; JS: Jitter and Shimmer; RBH: Roughness – Breathiness – Hoarseness; SAEs: serious adverse events; SCCGL: squamous cell carcinoma of glottic larynx; SPR: Singing Power Ratio; VHI: voice handicap index

*: Even if done on the same day, patient enrollment shall be done after the completion of the necessary documentation

#: VHI at 6 weeks after the end of treatment (regardless of study arm) will be assessed to evaluate the need for phoniatric rehabilitation, but <u>not</u> as a study-specific outcome measure (i.e. primary endpoint) to avoid lead time bias. Patients with a VHI > 14 will be recommended to undergo a phoniatric rehabilitation, but <u>the examiner should not try to persuade the patient</u>. Similarly, <u>any VHI >14 at any follow-up time point</u> or <u>patients' request</u> justifies the need for phoniatric rehabilitation, which will be recommended to the patients regardless of study arm. No need to record the VHI in the CRFs.

¥: The baseline toxicity scores and smoking status may be documented after the accrual but it must be done before the start of trial treatment.

Ψ: If clinically indicated

∇: The treatment shall start within 28 days post-accrual

9.2 Assessments of outcomes

The time points of each assessment are provided in the summary flow-charts above.

9.2.1 Assessment of primary outcome

The VHI[31] allows the person in question to make a subjective assessment of their own vocal problem. It is a questionnaire with 30 items (will be provided in different languages apart from the protocol). Using this questionnaire, the affected people can describe the degree to which their voice restricts their everyday life. The higher the score (0-120), the greater is their subjectively experienced handicap (score 0-14 = no; 15-28 = mild; 29-50 = moderate; 51-120 = severe handicap). The freeware open-source software DigitalVHI by Christian Herbst[32] will be used to record the VHI by each visit. DigitalVHI only generates pdf reports per VHI assessments and does not store any data locally or on a server.

9.2.2 Assessment of secondary outcomes

- Perceptual impression of the voice via **Roughness – Breathiness – Hoarseness (RBH)**: The perceptual impression of a vocal signal remains the gold standard in phoniatric diagnosis[33]. By means of reading the phonetically balanced text "The Rainbow Passage" in English and "Die Sonne und der Wind" in German, French and Italian (versions in different languages provided in Appendix C), the speaking voice is assessed by the investigator according to the parameters roughness – breathiness – hoarseness (RBH), using the scale of 0: normal, 1: mild, 2: moderate, 3: severe. In this study, the assessment will be carried out blind.

Quantitative characteristics of voice by means of;

- **Jitter and shimmer (JS)** are regarded as objective, quantitative characteristics of voice quality[34]. They describe the variations in the fundamental note of a vocal signal. Jitter is the variation of the cycle-to-cycle frequency in held vowels (in Hz or. %, norm = 0-3%), shimmer is the cycle-to-cycle variation in the amplitude of held vowels (in dB, or. %, norm = 0-20%). In this study, the assessment will be carried out blind.
- **Glottal-to-Noise Excitation Ratio (GNE)** serves to describe the relationship of the voice signal to the noise signal (norm = 1-0) and is also a quantitative characteristic of voice quality. Jitter, shimmer and GNE correspond to the perceptual characteristics roughness – breathiness – hoarseness[35,36]. In order to carry out JS and GNE, the test subjects hold the vowel /a/ *mezzoforte* for at least 5 seconds at their average speaking pitch. The vocal samples will be recorded and subsequently evaluated using the open source freeware software Praat (http://www.fon.hum.uva.nl/praat/ by Paul Boersma and David Weenink - Phonetic Sciences, University of Amsterdam Spuistraat 210 1012VT Amsterdam, Netherlands). In this study, the assessment will be carried out blind.
- **Singing Power Ratio (SPR):** The 'brilliant' sound of a voice is characterized in acoustic terms by a high degree of acoustic energy above 2 kHz. This can be calculated with the aid of the SPR[37,38]. In the Fast-Fourier-Transformation, the amplitudes of the highest peaks between 2 and 4 kHz and of the highest peaks between 0 and 2 kHz are determined using a vocal sample and the one subtracted from the other. The lower the SPR, the more "sonorous" is the voice. For this, the test subjects hold the vowel /a/ mezzoforte for at least 5 seconds at their average speaking pitch. The vocal samples will be recorded and subsequently evaluated using Praat. In this study, the assessment will be carried out blind.

Toxicity and Oncological Endpoints

- **Loco-regional control** of SCCGL: Loco-regional control is defined as the time between randomization and biopsy-proven HNSCC recurrence at the head and neck region. Patients without any loco-regional failure until the cut-off point will be censored at the last assessment date.
- **Treatment toxicity** at 2 and 5 years based on CTCAE v.5.0. The list of possible treatment-associated toxicities is provided in the Appendix A. Patients will not be further assessed for toxicity in case of a loco-regional and/or distant tumor recurrence.

9.2.3 Assessment of safety outcomes

Although the safety endpoints will be reported only at two time points (2 and 5 years), the toxicity will be systematically assessed during and after the treatment in a more frequent schedule: during the treatment, every 3 months until 24 months, and every 6 months between the 24th and 60th months. CTCAE v.5.0 will be used for the documentation of possible toxicities listed in Appendix A.

Adverse events (AE) of grades 1 to 2 (CTCAE v.5.0) between the recruitment and before the 6th month visit do not need to be reported in the study-specific documents and will be managed according to institutional local standards. Any AE of grade 3 and above is considered as a serious adverse event

(SAE) and shall be reported according to Section 10.

9.2.4 Assessments in participants who prematurely stop the study

If patients are withdrawn because of an AE, they will undergo physical examination and additional tests (e.g. laboratory testing) according to the nature of the AE. Unless the AE causes a contraindication for the planned treatment, it will be performed as planned according to tumor board decision. A last examination for possible (additional) toxicities shall be carried out. The follow-up period ends after this last examination or when patients are dismissed from the hospital after the treatment of the AE, whichever happens last.

9.3 Procedures at each visit

Procedures at each visit are described under Section 9.1 in Table 1.

10. SAFETY

An Adverse Event (**AE**) is any untoward medical occurrence in a patient or a clinical investigation subject which does not necessarily have a causal relationship with the trial procedure. An AE can therefore be any unfavorable or unintended finding, symptom, or disease temporally associated with a trial procedure, whether or not related to it.

Severity: The term "severe" is often used to describe the intensity (severity) of a specific event (as in mild, moderate or severe, or as described in CTCAE v.5.0 grades); the event itself, however, may be of relative minor medical significance (such as severe headache). This is not the same as "serious," which is based on patient/event outcome or action criteria usually associated with events that pose a threat to patient's life or functioning. Seriousness (not severity) serves as a guide for defining regulatory reporting obligations.

SAE: A **Serious Adverse Event** is defined as any untoward medical occurrence or effect in a patient, whether or not considered related to the protocol treatment, that:

- results in death;
- is life-threatening (i.e. an event in which the subject was at risk of death at the time of event; it does not refer to an event which hypothetically might have caused death if it was more severe);
- requires inpatient hospitalization or prolongation of existing patient hospitalization;
- results in persistent or significant disability or incapacity;
- is a congenital anomaly or birth defect;
- is a medically important event or reaction.

Medical and scientific judgment should be exercised in deciding whether other situations should be considered serious such as important medical events that might not be immediately life-threatening or result in death or hospitalization but might jeopardize the patient or might require intervention to prevent one of the other outcomes listed in the definition above.

Inpatient hospitalization: a hospital stay equal to, or greater than, 24 hours.

Second primary malignancy is one unrelated to the treatment of a previous malignancy (and is not a metastasis from the previous malignancy).

Secondary malignancy is a cancer caused by treatment for a previous malignancy (e.g., treatment with investigational agent/intervention, radiation or chemotherapy). A secondary malignancy is not considered a metastasis of the previous malignancy.

10.1 Exceptions

The following situations do not need to be reported as SAEs:

- Elective hospitalization for pre-existing conditions that have not been exacerbated by trial treatment.
- A hospitalization which was planned before the patient consented for study participation and where admission did not take longer than anticipated.
- A hospitalization planned for protocol related treatment or protocol related procedure as per institutional standard timelines.

- Social and/or convenience admission to a hospital.
- Medical or surgical procedure (e.g. endoscopy, appendectomy); the condition that leads to the procedure is already an (S)AE.
- Situations where an untoward medical occurrence did not occur (palliative care, rehabilitation, overdose without occurrence of an adverse event).
- Anticipated day-to-day fluctuations of pre-existing disease(s) or condition(s) present or detected at the start of the study that do not worsen.

Clinical events related to the primary cancer being studied or to the primary cancer progression are not to be reported as SAEs, even if they meet any of the seriousness criteria from the standard SAE definition, **unless** the event is more severe than expected and therefore the investigator considers that their clinical significance deserves reporting.

10.2 Severity assessment

The severity of all AEs (serious and non-serious) in this trial should be graded using CTCAE v.5.0.

10.3 Causality assessment

The investigator is obligated to assess the relationship between protocol treatment and the occurrence of each SAE following the definitions in the following table.

Table 5: Causality assessment for SAEs

Relationship to the protocol treatment	Description
Not related	There is clearly no reasonable possibility that the protocol treatment caused the event.
Unlikely	Any assessable reaction that does not fulfil the below conditions
Possibly	Temporal relationship, but other cause also possible
Probably	Temporal relationship, condition improved after dechallenge, no other cause evident
Definitely	Clearly related to intervention, temporal relationship, improvement of the condition after dechallenge, recurrence after rechallenge, or other cause of treatment cause

The investigator will use clinical judgment to determine the relationship. Alternative causes, such as natural history of the underlying diseases, medical history, concurrent conditions, concomitant therapy, other risk factors, and the temporal relationship of the event to the protocol treatment will be considered and investigated.

The decision will be recorded on the SAE form and if necessary the reason for the decision will also be recorded.

10.4 Expectedness assessment

The expectedness assessment is the responsibility of the sponsor of the study. The expectedness assessment will be performed against the following documents: study protocol, patient information sheet / informed consent.

10.5 SAE Reporting Procedure

For the time period beginning when the consent form is signed and the patient is registered in the study database, any SAE, or follow-up to a SAE, including death due to any cause, that occurs to any subject must be reported within 24 hours to the Sponsor **if is the result of a protocol-specified intervention/procedure.**

- All SAEs occurring from the time a subject is randomized until 90 days after last protocol treatment and;
- any SAE that occurs outside of the SAE detection period (after the 90-days period), if it is considered to have a reasonable possibility to be related to the protocol treatment or study

participation.

- Any secondary malignancy should also be reported in expedited way on a SAE form with the appropriate seriousness criteria.

All reporting must be done by the local principal investigator or authorized staff member (i.e. on the signature list) to confirm the accuracy of the report.

All SAE data must be collected on the study-specific SAE form.

All SAEs must be reported immediately and no later than 24 hours from the time the investigator or staff became aware of the event via fax (+41 31 632 85 67) using the SAE reporting form provided by the sponsor-investigator (exception for fax: site of the sponsor-investigator).

All SAE-related information needs to be provided in English.

All additional documents in local language must be accompanied by a translation in English, or the relevant information must be summarized in a follow-up SAE report form.

The investigator shall record all SAEs on the SAE sub-page of the eCRF.

The sponsor-investigator will re-evaluate the SAE and return the form to the site as required.

Recurrent episodes, complications, or progression of the initial SAE must be reported as follow-up to the original episode, regardless of when they occur. This report must be submitted within 24 hours of the investigator receiving the follow-up information. An SAE that is considered completely unrelated to a previously reported one should be reported separately as a new event.

10.6 Reporting to the Ethics Committee

The sponsor-investigator reports all SAEs for which it cannot be excluded that the events are attributable to the interventions under investigation to the to the coordinating ethics committee within 15 days. If the SAE occurred at one of the participating trial sites, the sponsor-investigator shall report the event also to the responsible local ethics committee concerned within the same period.

10.7 Reporting and Handling of Pregnancies

Pregnancy is an exclusion criterion for the study. However, if a pregnancy is diagnosed after the accrual (during the treatment or within the first 30 days of the follow-up phase), the continuation and discontinuation of the pregnancy (and the treatment) will be discussed with the patient. Any pregnancy during the treatment phase of the study and within 30 days after the study intervention will be reported to the sponsor-Investigator using the specific pregnancy reporting form within 24 hours. The pregnant partner of the patient participating in this trial will be asked for consent to follow-up the course and outcome of the pregnancy, to document and report any abnormal outcome regarding the mother or the child.

10.8 Periodic reporting of safety

An annual safety report is submitted once a year by the sponsor-investigator to all Ethics Committees involved.

11. STATISTICAL METHODS

11.1 Hypothesis and Determination of Sample Size

The sample size calculation is based on the primary outcome, the VHI at 6 to 24 months (averaged). Based on the literature, we consider the VHI to be comparable between TLM and traditional whole larynx radiotherapy. Therefore, the hypothesis is that there is a difference between TLM and SVCI in regard to VHI. Al-Mamgani et al.[12] reported standard deviations for VHI ranging from 2 to 10 score points. A difference of 8 points between the two groups is regarded as clinically relevant and a conservative standard deviation of 8 points is assumed. Based on a two-sample means test, 34 patients (17 per group) are needed to detect a difference in VHI at a two-sided alpha-level of 0.05 with a power of 80%.

This sample size calculation is conservative. In the analysis, the average change from baseline to the four time points (6, 12, 18, and 24 months) will be modelled and additionally adjusted for the baseline VHI to yield more power.

No statistical criteria for the termination of the trial is defined. No interim analysis for futility or safety will be performed.

11.2 Randomization and Stratification

A probabilistic minimization technique will be used for random treatment allocation between the two treatment arms using a 1:1 ratio. The computer chooses a treatment dynamically, based on the tumor stage (Stage 0 vs. I), and VHI at baseline (<34 vs. ≥34) as two predefined stratification factors.

Allocation will be done via a dedicated website within the clinical trial management system also containing the electronic case report forms. Only system administrators who are otherwise not involved in the trial will have access to the algorithm and stored lists during the recruitment period. Investigators receive the allocation only after registration of a patient. The underlying randomization lists and details of the minimization algorithm will not be disclosed but kept securely at CTU Bern. All these measures will help to ensure concealment of allocation.

11.3 Planned Analyses

A detailed description of data preparation and analysis will be elaborated in a statistical analysis plan (SAP).

11.3.1 Datasets to be analyzed, analysis populations

The primary analysis will follow the intention-to-treat (ITT) principle, i.e. all patients will be analyzed in the group they were originally assigned to at randomization regardless of any protocol violation.

In the per-protocol (PP) analysis, only patients that adhered to the protocol will be evaluated. Patients not receiving the allocated treatment or violating any of the inclusion criteria will be excluded from this analysis set.

11.3.2 Primary Analysis

The primary analysis will be based on the ITT patient set. Patient characteristics will be presented in a table, separate by treatment group, as number and percentage, mean and standard deviation, or median and interquartile range, as appropriate.

VHI collected over 24 months will be analyzed using a repeated-measures, linear mixed-effects model with a random intercept for the patients and fixed effects for the baseline value, the treatment group, the time points (categorical), interaction terms between treatment group and time points, and randomization stratification factors. We will present the mean VHI difference between the two groups with associated 95% confidence intervals and p-values over all time points (primary outcome) as well as separate for each time point (secondary outcomes) from this model.

JS, GNE, and SPR are measured on a continuous scale and, therefore, will be analyzed using the same approach as for VHI. Jitter and shimmer are expected to show a log-normal distribution and will therefore be log-transformed before the analysis. If the distributions of the other two outcomes also severely deviate from a normal distribution, the measures will be transformed in an adequate way, e.g. by log-transformation. If normality cannot be achieved, measures will be analyzed using a non-parametric Wilcoxon rank sum test, separate for each time point.

Roughness, breathiness, and hoarseness are each assessed on an ordinal scale ranging from 0 to 3. These measures will be dichotomized into 2/3 versus 0/1 and analyzed using mixed-effects logistic regression with a random intercept for the patients and fixed effects for treatment group, the time points (categorical), interaction terms between treatment group and time points, and randomization stratification factors. We will present the odds ratio with associated 95% confidence intervals and p-values over all time points as well as separate for each time point from this model.

The time-to-event outcome loco-regional control will be evaluated using Kaplan-Meier curves and a Cox model adjusted for the randomization stratification factors.

Treatment toxicity at 2 and 5 years will be summarized descriptively for each group, showing the overall number of events as well as number and percentage of patients with events.

Patients will be censored for toxicity assessment at the time of loco-regional and/or distant tumor recurrence.

11.3.3 Secondary Analyses

In a secondary analysis, we will analyze all outcomes using the PP patient set. Moreover, we will perform subgroup analyses according to tumor stage (stage 0 vs. I), and VHI at baseline (<34 vs. ≥34).

For RBH, we will also consider a proportional odds logistic regression of the original ordinal outcome. Since the assessment is qualitative and performed by different raters, we will also test for heterogeneity between the raters and include a random intercept for raters in logistic models, if necessary.

Toxicities in both arms will be described and compared with TAME[39–41] methodology.

Further secondary analyses may be described in the SAP.

11.3.4 Deviation(s) from the original statistical plan

Any deviation from the planned analysis, the rational therefore and the description of the deviation will be reported in the final study report and mentioned in publications of study results.

11.4 Handling of missing data and drop-outs

We expect that all randomized patients have complete baseline data. All patients that have at least one outcome assessment can be considered in repeated-measures analyses. Models will implicitly correct for missing data based on the missing at random mechanism. If there are patients with no outcome data at all, we will perform multiple imputation. Details will be described in the SAP. For the time-to-event analysis, patient drop-outs will be accounted for by censoring.

12. QUALITY ASSURANCE AND CONTROL

12.1 Data handling and record keeping / archiving

The investigators will maintain appropriate medical and research records for this trial, in compliance with ICH-GCP (E6) and regulatory and institutional requirements for the protection of confidentiality of subjects. The principal investigator, sub-investigator, and clinical research nurses or coordinators will have access to the records.

The principal investigators will permit authorized representatives of the Sponsor and regulatory agencies to examine (and when required by applicable law, to copy) clinical records for the purposes of quality assurance reviews, audits, and evaluation of the study safety and progress.

12.1.1 Case Report Forms

The CRFs will be electronic (eCRF). All data requested on the CRFs must be recorded and the recorded data should be consistent with the source documents or the discrepancies should be explained. The Investigator should ensure the accuracy, completeness, and timeliness of the data reported in the CRF and all other required reports. Generally, the CRFs should be completed within one week of completion of a patient visit.

12.1.2 Specification of source documents

Source documents must be available at the site to document the existence of the study participants and must include the original documents relating to the study, as well as the medical treatment and medical history of the participant. Where source documents for specific entries in the CRF are not available, this must be explicitly documented in a note to file. Any data recorded directly in the CRF will be considered as source data. Any change or correction to source data should be dated, initialed, and explained (if necessary) and should not obscure the original entry. The use of worksheets is allowed if the copies of the templates are documented in the trial master file (TMF). The used worksheets must be kept with the patient charts.

For all data captured in the CRF, the location of the source should be documented on a list of source documents, which will be stored in the investigator site file at each study site.

12.1.3 Record keeping / archiving

Essential documents (written and electronic), including images and radiotherapy plans must be retained for a period of at least 10 years from the completion or premature termination of the trial. The investigators should take measures to prevent accidental or premature destruction of these documents.

12.2 Data management

12.2.1 Data Management System

The CRFs in this trial are implemented electronically using a dedicated electronic data capturing (EDC) system (secuTrial®). The EDC system is activated for the trial only after successfully passing a formal test procedure. All data entered in the CRFs are stored on a Linux server in a dedicated Oracle database.

Responsibility for hosting the EDC system and the database lies with Inselspital Bern.

12.2.2 Data security, access and back-up

The server hosting the EDC system and the database is kept in a locked server-room. Only the system administrators have direct access to the server. A role concept with personal passwords (site investigator, statistician, monitor, administrator etc.) regulates permission for each user to use the system and database as he/she requires.

All data entered into the CRFs are transferred to the database using Secure Sockets Layer (SSL) encryption. Each data point has attributes attached to it identifying the user who entered it with the exact time and date. Retrospective alterations of data in the database are recorded in an audit table. Time, table, data field and altered value, and the person are recorded (audit trail). A multi-level back-up system is implemented.

12.2.3 Data transfer for radiotherapy data and sound recordings

The radiotherapy plans will be transferred to the RT-QA group's database via a HFG-conform server, which will be defined by the RT-QA group. After the data transfer takes place (i.e. downloaded to the RT-QA group's database), the files on the server will be deleted. The same system will be used to transfer the sound files recorded for the secondary study endpoints concerning voice quality.

12.2.4 Analysis and archiving

At final analysis, data files will be extracted from the database into statistical packages to be analyzed. The status of the database at this time is recorded in special archive tables.

The study database with all archive tables will be securely stored by Inselspital Bern. The sponsor-investigator also keeps the TMF and interim and final reports both in electronic and in hard copy form for at least 10 years.

12.2.5 Electronic and central data validation

Data can be entered into the database only after a check of completeness and plausibility. Furthermore, selected data points are cross-checked for plausibility with previously entered data for that participant. In addition, central data reviews will be performed on a regular basis to ensure completeness of the data collected and accuracy of the primary outcome data.

12.3 Monitoring

For quality control of the study conduct and data retrieval, all study sites will be visited on-site by appropriately trained and qualified monitors. Any findings and comments will be documented in site visit reports and communicated to the local PI and to the sponsor as applicable. Investigators at the participating study sites will support the monitor in his/her activities. Prior to study start (first participant enrolled) a plan detailing all monitoring-related procedures will be developed.

All source data and relevant documents will be accessible to monitors and questions of monitors are answered during site visits.

12.4 Audits and Inspections

Source data/documents must be available to audits by the sponsor or designee or to inspections by health authorities.

A regulatory authority or ethics committee may wish to conduct an inspection (during the study or after its completion). If an inspection is requested, the investigator must inform the sponsor immediately that this request has been made. The investigators at the participating sites will support the inspectors in their activities.

The sponsor may exclude participating sites/investigators from further participation in the study because of fraud or non-compliance with the study protocol, ICH-GCP guidelines, or applicable laws.

Study sites or investigators may stop recruiting patients to this study when the investigator deems inclusion of patients into this study to be no longer ethical for medical or organizational reasons. In this case, the investigator should give detailed reasons to the Sponsor.

12.5 Confidentiality, Data Protection

The investigator ensures anonymity of the patients; patients will not be identified by names in any documents. Signed informed consent forms and patient enrollment log will be kept strictly confidential to enable patient identification at the site.

13. PUBLICATION AND DISSEMINATION POLICY

The results will be published in the name of the VoiceS trial in a peer reviewed international journal on behalf of all collaborators. All presentations and publications, including abstracts, relating to the trial must be authorized by the VoiceS trial steering committee (all co-investigators listed in the protocol). Participating centers should ask for the approval of the trial steering committee to use any data related to the patients registered in the trial.

14. FUNDING AND SUPPORT

14.1 Funding

The study is to be funded completely by external grants. The total cost is estimated to be CHF 202’724 (CTU Bern: CHF 86’593; RT-QA and data transfer: CHF 15’645; phoniatric assessments: CHF: 30’486; patient fees: CHF 68’000; Ethics committee costs [based on tariffs of ethics committee Bern 2018]: CHF 2’000).

14.2 Other Support

Not applicable/relevant

15. INSURANCE

Insurance will be provided by the University Hospital of Bern, Inselspital. A copy of the certificate is filed in each investigator site file and the trial master file.

16. REFERENCES

1. Mo HL, Li J, Yang X, et al. Transoral laser microsurgery versus radiotherapy for T1 glottic carcinoma: a systematic review and meta-analysis. *Lasers Med Sci*. 2017;32(2):461-467. doi:10.1007/s10103-016-2103-8
2. Warner L, Chudasama J, Kelly CG, et al. Radiotherapy versus open surgery versus endolaryngeal surgery (with or without laser) for early laryngeal squamous cell cancer. *Cochrane database Syst Rev*. 2014;12(12):CD002027. doi:10.1002/14651858.CD002027.pub2
3. Abdurehim Y, Hua Z, Yasin Y, Xukurhan A, Imam I, Yuqin F. Transoral laser surgery versus radiotherapy: systematic review and meta-analysis for treatment options of T1a glottic cancer. *Head Neck*. 2012;34(1):23-33. doi:10.1002/hed.21686
4. Greulich MT, Parker NP, Lee P, Merati AL, Misono S. Voice outcomes following radiation versus laser microsurgery for T1 glottic carcinoma: systematic review and meta-analysis. *Otolaryngol Head Neck Surg*. 2015;152(5):811-819. doi:10.1177/0194599815577103
5. Huang G, Luo M, Zhang J, Liu H. Laser surgery versus radiotherapy for T1a glottic carcinoma: a meta-analysis of oncologic outcomes. *Acta Otolaryngol*. 2017;137(11):1204-1209. doi:10.1080/00016489.2017.1353706
6. Aaltonen L-M, Rautiainen N, Sellman J, et al. Voice quality after treatment of early vocal cord cancer: a randomized trial comparing laser surgery with radiation therapy. *Int J Radiat Oncol Biol Phys*. 2014;90(2):255-260. doi:10.1016/j.ijrobp.2014.06.032
7. Osman SOS, Astreinidou E, de Boer HCJ, et al. IMRT for image-guided single vocal cord irradiation. *Int J Radiat Oncol Biol Phys*. 2012;82(2):989-997. doi:10.1016/j.ijrobp.2010.12.022
8. Levendag PC, Teguh DN, Keskin-Cambay F, et al. Single vocal cord irradiation: a competitive treatment strategy in early glottic cancer. *Radiother Oncol*. 2011;101(3):415-419. doi:10.1016/j.radonc.2011.05.026
9. Osman SOS, de Boer HCJ, Astreinidou E, Gangsaas A, Heijmen BJM, Levendag PC. On-line cone beam CT image guidance for vocal cord tumor targeting. *Radiother Oncol*. 2009;93(1):8-13. doi:10.1016/j.radonc.2009.05.015
10. Osman SOS, de Boer HCJ, Heijmen BJM, Levendag PC. Four-dimensional CT analysis of vocal cords mobility for highly focused single vocal cord irradiation. *Radiother Oncol*. 2008;89(1):19-27. doi:10.1016/j.radonc.2008.05.016
11. Kwa SLS, Al-Mamgani A, Osman SOS, Gangsaas A, Levendag PC, Heijmen BJM. Inter- and Intrafraction Target Motion in Highly Focused Single Vocal Cord Irradiation of T1a Larynx Cancer Patients. *Int J Radiat Oncol Biol Phys*. 2015;93(1):190-195. doi:10.1016/j.ijrobp.2015.04.049
12. Al-Mamgani A, Kwa SLS, Tans L, et al. Single Vocal Cord Irradiation: Image Guided Intensity Modulated Hypofractionated Radiation Therapy for T1a Glottic Cancer: Early Clinical Results. *Int J Radiat Oncol Biol Phys*. 2015;93(2):337-343. doi:10.1016/j.ijrobp.2015.06.016
13. Remacle M, Eckel HE, Antonelli A, et al. Endoscopic cordectomy. A proposal for a classification by the Working Committee, European Laryngological Society. *Eur Arch Otorhinolaryngol*. 2000;257(4):227-231. http://www.ncbi.nlm.nih.gov/pubmed/10867840.
14. Mastronikolis N, Papadas T, Goumas P, et al. Head and neck: Laryngeal tumors: an overview. *Atlas Genet Cytogenet Oncol Haematol*. 2011;13(11):888-893. doi:10.4267/2042/44625
15. Lyhne NM, Johansen J, Kristensen CA, et al. Pattern of failure in 5001 patients treated for glottic squamous cell carcinoma with curative intent - A population based study from the DAHANCA group. *Radiother Oncol*. 2016;118(2):257-266. doi:10.1016/j.radonc.2016.02.006
16. Grégoire V, Lefebvre J-L, Licitra L, Felip E, EHNS-ESMO-ESTRO Guidelines Working Group. Squamous cell carcinoma of the head and neck: EHNS-ESMO-ESTRO Clinical Practice Guidelines for diagnosis, treatment and follow-up. *Ann Oncol Off J Eur Soc Med Oncol*. 2010;21 Suppl 5(Supplement 5):v184-6. doi:10.1093/annonc/mdq185
17. "National Comprehensive Cancer Network." National Comprehensive Cancer Network Guidelines for Head and Neck Cancers (version 2.2018). http://www.nccn.org/professionals/physician_gls/pdf/head-and-neck.pdf. Published 2018. Accessed December 5, 2018.
18. Goor KM, Peeters AJGE, Mahieu HF, et al. Cordectomy by CO2 laser or radiotherapy for small T1a glottic carcinomas: costs, local control, survival, quality of life, and voice quality. *Head Neck*.

2007;29(2):128-136. doi:10.1002/hed.20500

19. Loughran S, Calder N, MacGregor FB, Carding P, MacKenzie K. Quality of life and voice following endoscopic resection or radiotherapy for early glottic cancer. *Clin Otolaryngol*. 2005;30(1):42-47. doi:10.1111/j.1365-2273.2004.00919.x

20. Gandhi S, Gupta S, Rajopadhye G. A comparison of phonatory outcome between trans-oral CO2 Laser cordectomy and radiotherapy in T1 glottic cancer. *Eur Arch Otorhinolaryngol*. 2018;275(11):2783-2786. doi:10.1007/s00405-018-5152-8

21. Dinapoli N, Parrilla C, Galli J, et al. Multidisciplinary approach in the treatment of T1 glottic cancer. The role of patient preference in a homogenous patient population. *Strahlenther Onkol*. 2010;186(11):607-613. doi:10.1007/s00066-010-2142-1

22. Jotic A, Stankovic P, Jesic S, Milovanovic J, Stojanovic M, Djukic V. Voice quality after treatment of early glottic carcinoma. *J Voice*. 2012;26(3):381-389. doi:10.1016/j.jvoice.2011.04.004

23. Tomifuji M, Araki K, Niwa K, et al. Comparison of voice quality after laser cordectomy with that after radiotherapy or chemoradiotherapy for early glottic carcinoma. *ORL J Otorhinolaryngol Relat Spec*. 2013;75(1):18-26. doi:10.1159/000346934

24. Dixon LM, Douglas CM, Shaukat SI, et al. Conventional fractionation should not be the standard of care for T2 glottic cancer. *Radiat Oncol*. 2017;12(1):178. doi:10.1186/s13014-017-0915-8

25. Dindo D, Demartines N, Clavien P-A. Classification of surgical complications: a new proposal with evaluation in a cohort of 6336 patients and results of a survey. *Ann Surg*. 2004;240(2):205-213. doi:10.1097/01.sla.0000133083.54934.ae

26. Elicin O, Terribilini D, Shelan M, et al. Primary tumor volume delineation in head and neck cancer: missing the tip of the iceberg? *Radiat Oncol*. 2017;12(1):102. doi:10.1186/s13014-017-0838-4

27. Brouwer CL, Steenbakkers RJHM, Bourhis J, et al. CT-based delineation of organs at risk in the head and neck region: DAHANCA, EORTC, GORTEC, HKNPCSG, NCIC CTG, NCRI, NRG Oncology and TROG consensus guidelines. *Radiother Oncol*. 2015;117(1):83-90. doi:10.1016/j.radonc.2015.07.041

28. Christianen MEMC, Langendijk J a., Westerlaan HE, Van De Water T a., Bijl HP. Delineation of organs at risk involved in swallowing for radiotherapy treatment planning. *Radiother Oncol*. 2011;101(3):394-402. doi:10.1016/j.radonc.2011.05.015

29. van Asselen B, Raaijmakers CPJ, Lagendijk JJW, Terhaard CHJ. Intrafraction motions of the larynx during radiotherapy. *Int J Radiat Oncol Biol Phys*. 2003;56(2):384-390. doi:10.1016/S0360-3016(02)04572-8

30. Alaei P, Spezi E. Imaging dose from cone beam computed tomography in radiation therapy. *Phys Med*. 2015;31(7):647-658. doi:10.1016/j.ejmp.2015.06.003

31. Jacobson BH, Johnson A, Grywalski C, Silbergleit A, Jacobson G, Benninger MS NC. The voice handicap index: development and validation. *Am J Speech Lang Pathol*. 1997;6(3):66–70.

32. Herbst CT, Oh J, Vydrová J, Švec JG. DigitalVHI--a freeware open-source software application to capture the Voice Handicap Index and other questionnaire data in various languages. *Logoped Phoniatr Vocol*. 2015;40(2):72-76. doi:10.3109/14015439.2013.830769

33. Dejonckere PH, Bradley P, Clemente P, et al. A basic protocol for functional assessment of voice pathology, especially for investigating the efficacy of (phonosurgical) treatments and evaluating new assessment techniques: Guideline elaborated by the Committee on Phoniatrics of the European Laryngolo. *Eur Arch Oto-Rhino-Laryngology*. 2001;258(2):77-82. doi:10.1007/s004050000299

34. Brockmann M, Drinnan MJ, Storck C, Carding PN. Reliable jitter and shimmer measurements in voice clinics: the relevance of vowel, gender, vocal intensity, and fundamental frequency effects in a typical clinical task. *J Voice*. 2011;25(1):44-53. doi:10.1016/j.jvoice.2009.07.002

35. Michaelis D, Gramss T, Strube H. Glottal to Noise Excitation Ratio - a new measure for describing pathological voices. *Acustica*. 1997;83:700-706.

36. Teixera J, Fernandes P. Jitter, Shimmer and HNR classification within gender, tones and vowels in healthy voices. *Procedia Technol*. 2014;16:1228-1237.

37. Omori K, Kacker A, Carroll LM, Riley WD, Blaugrund SM. Singing power ratio: quantitative evaluation of singing voice quality. *J Voice*. 1996;10(3):228-235. http://www.ncbi.nlm.nih.gov/pubmed/8865093.

38. Watts C, Barnes-Burroughs K, Estis J, Blanton D. The singing power ratio as an objective measure of singing voice quality in untrained talented and nontalented singers. *J Voice*. 2006;20(1):82-88. doi:10.1016/j.jvoice.2004.12.003

39. Trotti A, Pajak TF, Gwede CK, et al. TAME: development of a new method for summarising adverse events of cancer treatment by the Radiation Therapy Oncology Group. *Lancet Oncol*. 2007;8(7):613-624. doi:10.1016/S1470-2045(07)70144-4

40. Gillison ML, Trotti AM, Harris J, et al. Radiotherapy plus cetuximab or cisplatin in human papillomavirus-positive oropharyngeal cancer (NRG Oncology RTOG 1016): a randomised, multicentre, non-inferiority trial. *Lancet (London, England)*. 2019;393(10166):40-50. doi:10.1016/S0140-6736(18)32779-X

41. Mehanna H, Robinson M, Hartley A, et al. Radiotherapy plus cisplatin or cetuximab in low-risk human papillomavirus-positive oropharyngeal cancer (De-ESCALaTE HPV): an open-label randomised controlled phase 3 trial. *Lancet (London, England)*. 2019;393(10166):51-60. doi:10.1016/S0140-6736(18)32752-1

17. APPENDICES

APPENDIX A: List of Possible Treatment-Related Toxicities (CTCAE v.5.0)

Tissue/Organ	Grade 1	Grade 2	Grade 3	Grade 4
**Skin Induration**	Mild induration, able to move skin parallel to plane (sliding) and perpendicular to skin (pinching up)	Moderate induration, able to slide skin, unable to pinch skin; instrumental ADL	Severe induration, unable to slide skin; limiting joint movement or orifice (e.g. mouth, anus); limiting self-care ADL	Generalized; associated with signs or symptoms of impaired breathing or feeding
**Skin Atrophy**	Covering <10% BSA; associated with telangiectasias or changes in skin color	Covering 10 - 30% BSA; associated with striae or adnexal structure loss	Covering >30% BSA; associated with ulceration	-
**Dysphagia**	Symptomatic, able to eat regular diet	Symptomatic and altered eating/swallowing	Severely altered eating/swallowing; tube feeding or total parenteral nutrition or hospitalization indicated	Life-threatening consequences; urgent intervention indicated
**Hoarseness**	Mild or intermittent voice change; fully understandable; self-resolves	Moderate or persistent voice changes; may require occasional repetition but understandable on telephone; medical evaluation indicated	Severe voice changes including predominantly whispered speech	-
**Laryngeal Stenosis**	Asymptomatic; clinical or diagnostic observations only; intervention not indicated	Symptomatic (e.g., noisy airway breathing), no respiratory distress; medical intervention indicated (e.g., steroids); limiting instrumental ADL	Limiting self-care ADL; stridor; endoscopic intervention indicated (e.g., stent, laser)	Life-threatening consequences; urgent intervention indicated
**Pharyngeal Stenosis**	Asymptomatic; clinical or diagnostic observations only; intervention not indicated	Symptomatic (e.g., noisy airway breathing), no respiratory distress; medical intervention indicated (e.g., steroids); limiting instrumental ADL	Limiting self-care ADL; stridor; endoscopic intervention indicated (e.g., stent, laser)	Life-threatening airway compromise; urgent intervention indicated (e.g., tracheotomy or intubation)
**Aspiration**	Asymptomatic; clinical or diagnostic observations only; intervention not indicated	Altered eating habits; coughing or choking episodes after eating or swallowing; medical intervention indicated (e.g., suction or oxygen)	Dyspnea and pneumonia symptoms (e.g., aspiration pneumonia); hospitalization indicated; unable to aliment orally	Life-threatening respiratory or hemodynamic compromise; intubation or urgent intervention indicated
**Pain**	Mild pain	Moderate pain limiting instrumental ADL	Severe pain limiting self-care ADL	-
**Hypothyroidism**	Asymptomatic; clinical or diagnostic	Symptomatic; thyroid replacement	Severe symptoms; limiting self-care	Life-threatening consequences;

	observations only; intervention not indicated	indicated; limiting instrumental ADL	ADL; hospitalization indicated	urgent intervention indicated
**Fatigue**	Fatigue relieved by rest	Fatigue not relieved by rest; limiting instrumental ADL	Fatigue not relieved by rest, limiting self-care ADL	-
**Peripheral Motor Neuropathy**	Asymptomatic; clinical or diagnostic observations only	Moderate symptoms; limiting instrumental ADL	Severe symptoms; limiting self-care ADL	Life-threatening consequences; urgent intervention indicated
**Peripheral Sensory Neuropathy**	Asymptomatic; clinical or diagnostic observations only	Moderate symptoms; limiting instrumental ADL	Severe symptoms; limiting self-care ADL	Life-threatening consequences; urgent intervention indicated

For all: 0 - no symptoms; 5 - death directly related to the particular effect

ADL: Activities of daily living

Instrumental ADL refer to preparing meals, shopping for groceries or clothes, using the telephone, managing money, etc.

Self-care ADL refer to bathing, dressing and undressing, feeding self, using the toilet, taking medications, and not bedridden.

APPENDIX B: Clavien-Dindo Classification of Surgical Complications

TABLE 1. Classification of Surgical Complications

Grade	Definition
Grade I	Any deviation from the normal postoperative course without the need for pharmacological treatment or surgical, endoscopic, and radiological interventions
	Allowed therapeutic regimens are: drugs as antiemetics, antipyretics, analgetics, diuretics, electrolytes, and physiotherapy. This grade also includes wound infections opened at the bedside
Grade II	Requiring pharmacological treatment with drugs other than such allowed for grade I complications
	Blood transfusions and total parenteral nutrition are also included
Grade III	Requiring surgical, endoscopic or radiological intervention
Grade IIIa	Intervention not under general anesthesia
Grade IIIb	Intervention under general anesthesia
Grade IV	Life-threatening complication (including CNS complications)* requiring IC/ICU management
Grade IVa	Single organ dysfunction (including dialysis)
Grade IVb	Multiorgan dysfunction
Grade V	Death of a patient
Suffix "d"	If the patient suffers from a complication at the time of discharge (see examples in Table 2), the suffix "d" (for "disability") is added to the respective grade of complication. This label indicates the need for a follow-up to fully evaluate the complication.

*Brain hemorrhage, ischemic stroke, subarrachnoidal bleeding, but excluding transient ischemic attacks.
CNS, central nervous system; IC, intermediate care; ICU, intensive care unit.

Dindo D, Demartines N, Clavien P-A. Classification of surgical complications: a new proposal with evaluation in a cohort of 6336 patients and results of a survey. Ann Surg. 2004;240(2):205–13.

APPENDIX C: "The Rainbow Passage" in English and "Die Sonne und der Wind" by Johann Gottfried Herder in Different Languages

# - The Rainbow Passage (English) -

When the sunlight strikes raindrops in the air, they act as a prism and form a rainbow. The rainbow is a division of white light into many beautiful colors. These take the shape of a long round arch, with its path high above, and its two ends apparently beyond the horizon. There is, according to legend, a boiling pot of gold at one end. People look, but no one ever finds it. When a man looks for something beyond his reach, his friends say he is looking for the pot of gold at the end of the rainbow.

Throughout the centuries people have explained the rainbow in various ways. Some have accepted it as a miracle without physical explanation. To the Hebrews it was a token that there would be no more universal floods. The Greeks used to imagine that it was a sign from the gods to foretell war or heavy rain. The Norsemen considered the rainbow as a bridge over which the gods passed from earth to their home in the sky.

(Articulation exercise including all the normal sounds of spoken English)

AV3F English Public Speaking (Hopkins)

Department of Translation Studies, University of Tampere

The Rainbow Passage can be found on page 127 of the 2nd edition of Grant Fairbanks' Voice and Articulation Drillbook (1960, pp. 124-139, New York: Harper & Row).

# - Die Sonne und der Wind (German) -

Einst stritten sich die Sonne und der Wind, wer von ihnen beiden der Stärkere sei, und man wurde sich einig, derjenige sollte dafür gelten, der einen Wanderer, den sie eben vor sich sahen, am ersten nötigen würde, seinen Mantel abzulegen. Sogleich begann der Wind zu stürmen; Regen und Hagelschauer unterstützten ihn. Der arme Wanderer jammerte und stöhnte; aber immer fester wickelte er sich in seinen Mantel ein und setzte seinen Weg fort, so gut er konnte. Jetzt kam die Reihe an die Sonne. Mit milder und sanfter Glut ließ sie ihre Strahlen herabfallen. Himmel und Erde wurden heiter; die Lüfte erwärmten sich. Der Wanderer vermochte den Mantel nicht länger auf seinen Schultern zu erdulden. Er warf ihn ab und erquickte sich im Schatten eines Baumes, während die Sonne sich ihres Sieges erfreute.

# - La brise et le soleil (French) -

La brise et le soleil se disputaient, chacun assurant qu'il était le plus fort, quand ils ont vu un voyageur qui s'avancait, enveloppé dans son manteau. Ils sont tombés d'accord que celui qui arriverait le premier à faire ôter son manteau au voyageur, serait regardé comme le plus fort. Alors la brise s'est mise à souffler de toute sa force, mais plus elle soufflait, plus le voyageur serrait son manteau autour de lui: et à la fin la brise a renoncé à le lui faire ôter. Alors le soleil a commencé à briller, et au bout d`un moment le voyageur, rechauffé, a ôté son manteau. Ainsi la brise a du reconnaître que le soleil était le plus fort des deux.

# - Il sole ed il vento (Italian) -

Una volta il sole ed il vento si disputarono per chi fosse il più forte di loro. All’improvviso videro passare un signore con un gran cappotto e decisero allora di sfidarsi: il più forte sarebbe stato colui che per primo fosse riuscito a far togliere il cappotto al signore. Il vento si mise a soffiare con tutta la sua forza, ma più forte soffiava, più il signore stringeva il cappotto. Così il vento si arrese. Il sole, dunque, cominciò a splendere e, poco dopo, il signore tolse il cappotto. A questo punto il vento dovette ammettere che il sole era il più forte dei due.
